# Processes of change in family therapies for anorexia nervosa: a systematic review and meta-synthesis of qualitative data

**DOI:** 10.1186/s40337-024-01037-5

**Published:** 2024-07-25

**Authors:** Sophie Cripps, Lucy Serpell, Matthew Pugh

**Affiliations:** Research Department of Clinical, Educational and Health Psychology, University College London, Gower Street, London, WC1E 6BT UK

**Keywords:** Anorexia nervosa, Family therapy, Qualitative research, Meta-synthesis, Families’ perspectives, Systematic review

## Abstract

**Objective:**

To synthesise young person and family member perspectives on processes of change in family therapy for anorexia nervosa (AN), including systemic family therapy and manualised family-based treatments, to obtain an understanding of what helps and hinders positive change.

**Method:**

A systematic search of the literature was conducted to identify qualitative studies focussing on experiences of therapeutic change within family therapies for AN from the perspectives of young people and their families. Fifteen studies met inclusion criteria and underwent quality appraisal following which they were synthesised using a meta-synthesis approach.

**Results:**

Six overarching themes were generated: “A holistic focus on the young person’s overall development”; “The therapeutic relationship as a vehicle for change”; “The therapist’s confinement to a script and its impact on emotional attunement”; “A disempowering therapeutic context”; “Externalisation of the eating disorder (ED)”; and “The importance of family involvement”. Positive change was helped by understanding and support given to the young person’s overall development including their psychological, emotional, social and physical wellbeing, positive therapeutic relationships, relational containment within the family system and externalising conversations in which young people felt seen and heard. Positive change was hindered by inflexibility in the treatment approach, counter-effects of externalisation, negative experiences of the therapist, a narrow focus on food-intake and weight, as well as the neglect of family difficulties, emotional experiences, and psychological factors.

**Conclusions:**

Positive change regarding the young person’s eating-related difficulties ensued in the context of positive relational changes between the young person, their family members, the therapist and treatment team, highlighting the significance of secure and trusting relationships. The findings of this review can be utilised by ED services to consider how they may adapt to the needs of young people and their families in order to improve treatment satisfaction, treatment outcomes, and in turn reduce risk for chronicity in AN.

## Abstract (plain language summary)

This review synthesises the views of young people and their family members regarding their perspectives of therapeutic change within family therapies for Anorexia Nervosa (AN), including both manualised eating disorder-focussed family therapy models (family-based treatment; FBT and AN-focussed family therapy; FT-AN), as well as systemic family therapy (SyFT), to understand which aspects of these treatment approaches are helpful versus hindering to recovery from an eating disorder (ED). Parental involvement was crucial in facilitating the restoration of physical health through the process of parents taking temporary responsibility for the young person’s eating behaviours until they can feed themselves again. However, treatment often failed to acknowledge and address the psychological and emotional difficulties that made the young person vulnerable to developing AN, as well as the psychological distress caused by increasing food-intake and weight. A positive therapeutic relationship in which families felt well supported by their therapist was important in providing containment during a time of familial strain and instability, yet there was a need for greater flexibility and individualisation within manualised ED-focussed family therapy approaches, particularly FBT. The findings highlight the importance of eliciting the young person’s voice to enhance their personal agency in treatment and the value of therapeutic space to improve family functioning and enhance family unity. Lastly, they illuminate the need for manualised ED-focussed family therapy models to allow space for the therapist to emotionally attune to young people and families in order to contain their experience of distress.

## Background

Characterised by excessive preoccupation with control over body weight and eating resulting in life-threatening physical effects, Anorexia Nervosa (AN) has significant emotional, social and relational implications for the individual and their family [1–3]. Whilst the onset is typically in adolescence, AN can continue into adulthood [4]. The aetiology of AN is complex and multifactorial [5]; research suggests an interaction of genetic risk with other factors including emotion dysregulation, anxiety, perfectionism, cognitive rigidity, and early feeding difficulties [6–9].

### Family therapies for anorexia nervosa

Family therapies are currently the first line treatment for AN in children and adolescents recommended by the National Institute for Health and Care Excellence (NICE) [10]. The development of eating disorder (ED) focussed family therapy models have been built on earlier approaches which were informed by Family Systems Theory. Family Systems Theory describes how family dynamics and processes can contribute to the development and, or maintenance of problems within the family system [11]. Since then, models of family therapy for AN have evolved and emphasise that families are a resource, rather than a treatment target [12].

Family therapy for AN was first developed into a manualised treatment named ‘family-based treatment’ (FBT) by Lock and Le Grange [13]. Since then, this manualised form of family therapy has been futher updated by Eisler and colleagues and is named ‘AN-focussed family therapy’ (FT-AN) [14]. These manualised treatment approaches were developed at the Maudsley, hence they may also be referred to as ‘the Maudsley method’. They are the most evaluated and widely used family therapies for adolescent AN.

Although there are subtle differences between FBT and FT-AN, they are conceptually similar and share certain fundamental principles [15]. Firstly, families are seen as holding important resources, which can be enhanced to help the young person recover; and linked closely with this is the understanding that families have not caused the illness. Secondly, they emphasise the importance of supporting parents to have a central role in managing their young person’s eating from the outset of treatment whilst also holding the longer term understanding that this will need to change through the process of recovery which requires the young person to reclaim developmentally appropriate individuation and independence. Both ED-focussed family therapy models externalise the ED from the outset, prioritise an initial focus on the restoration of food-intake and weight restoration, and subsequently shift focus on to adolescent and family developmental life cycle issues in the later stages of treatment.

While FBT and FT-AN are more similar than different, they vary somewhat in the number of phases described, their emphasis on engagement, the use of formulation, the raising of parental anxiety and the inclusion of individual sessions with the young person [16]. The original FBT method is outlined below, and its further development within FT-AN is subsequently described.

### Family-based treatment for anorexia nervosa (FBT)

FBT is a manualised outpatient three phase therapy with a behavioural and educative focus. Phase one focuses on refeeding to increase weight orchestrated by parents who are temporarily given responsibility for the individual’s eating and exercise patterns. Phase two focuses on gradually developing the young person’s independence by progressively returning responsibility for eating to the individual. When safe to do so, the focus is taken away from food to problem-solve family and psychosocial issues which interfere with weight restoration. Finally, phase three addresses remaining concerns related to adolescent development, including the re-establishment of healthy boundaries within the family system, and navigating approaching developmental challenges without reverting to ED behaviours. FBT has five key tenets [17]:

1. The therapist takes an agnostic stance, engaging the family in facilitating early behavioural change to improve the management of eating-related behaviours, rather than exploring or resolving aetiology.
2. The therapist uses externalising language encouraging the family to conceptualise AN as an “illness” which has “taken over” the young person.
3. The therapist takes a non-authoritarian therapeutic stance, viewing parents as experts in their family and assuming that giving parents responsibility for weight restoration enables reorganisation of the family to enhance parental effectiveness.
4. Therapists empower parents to orchestrate recovery. By not providing explicit instructions, parental confidence is built through allowing for struggle and self-reliance.
5. FBT utilises a pragmatic approach, adopting a firm initial focus on restoring physical health. Comorbid difficulties are not directly addressed in phase one to ensure that weight restoration is the primary focus and because many are assumed to resolve with eating and weight restoration.

### Anorexia Nervosa focussed family therapy (FT-AN)

FT-AN specifically emphasises the engagement of all family members from the outset of treatment, including the young person, and the use of formulation to ensure treatment is individually tailored [18, 19]; FT-AN is a phased treatment which includes (1) engagement and development of the therapeutic alliance; (2) helping the family to manage the eating disorder with specific emphasis on reframing parental feeding as care, not control; (3) exploring issues of individual and family development; (4) ending treatment, discussion of future plans and discharge [14]. The early phases of treatment have a strong focus on practically supporting young people and families to manage ED symptoms, gain weight (if required) and build skills around tolerating related distress. The content of sessions in the later phases is individualised to each family. Common themes include returning to independent eating, managing school and peer relationships, tolerating uncertainty, etc. The number and frequency of FT-AN sessions are not pre-determined, rather they are based on family need and clinical presentation.

### Systemic family therapy for anorexia nervosa (SyFT)

Systemic Family Therapy (SyFT) is also used in some settings [20]. SyFT is a less defined family therapy which is not delivered with adherence to a phased manual. SyFT does not have defined principles specifically related to the clinical problem (AN) but more so principles to guide the therapist’s thinking and questioning. SyFT focuses on relationships and making meaning of behaviours to promote change, whereas FBT and FT-AN have a more agnostic, behavioural and practical focus on symptom change. Similar to FBT and FT-AN, the SyFT therapist takes a non-blaming, collaborative stance and encourages parental agency and alliance, however they place greater focus on the family system [21]. Difficulties are conceptualised as arising from the interpersonal relationships, dynamics and narratives about a problem within a family system. Adopting a neutral stance, the therapist explores family patterns of beliefs and behaviours, seeking ways to enable the family to draw on their strengths and generate solutions. SyFT aims to enable family members to express and explore difficult thoughts and emotions safely, understand each other’s experiences and views, appreciate each others needs, and work together to make useful changes in their relationsips and lives [22]. Although there is not a specific emphasis on normalisation of eating or weight, the therapist helps the family address these issues when raised.

### Research on family therapies for anorexia nervosa

This section reviews qualitative and quantitative research which has focussed on young people and family member experiences of treatments for AN, as well as their mechanisms of change. A meta-synthesis of views about treatment from the perspectives of young people, parents and professionals underlined the central importance of the therapeutic relationship yet the difficulty in forming alliance due to disagreement about treatment targets and mutual distrust [1]. For therapists, the treatment target was normalisation of the young person’s eating and weight. However, young people wanted treatment to target their psychological and social functioning, as well as their family environment; and parents wanted treatment to explore the origins and causes of AN.

Another meta-synthesis focussed on FBT/SyFT found that whilst young people experienced extreme challenge relinquishing control of their eating, they also considered their caregivers’ involvement in this regard as one of the most important aspects of treatment [23]. However, they also appreciated the gradual restoration of autonomy and assistance with difficulties in family relationships. Moreover, whilst externalisation helped to reduce family criticism and increase praise, young people who engaged in FBT would have liked the causes of AN and other difficulties to have been addressed. Lastly, some young people would have liked individual sessions to address issues they did not feel comfortable discussing with family present.

One recent study which aimed to explore processes of change during FT-AN from the perspective of young people has suggested several factors to be key in promoting change: (1) emphasising engagement with all family members from the outset, (2) ensuring life outside the eating disorder is always brought into treatment sessions, (3) supporting the young person to understand their illness from a different perspective and recognise its impact, (4) creating a home and treatment environment in which the illness cannot be avoided and will be addressed [24].

Service-users emphasise that treatments are most helpful when they recognise the emotional impact of weight gain and address psychological as well as physical aspects of AN [1, 23, 25–27]. They highlight the perceived unhelpfulness of professionals’ conceptualisation of recovery as a pre-set target weight, rather than considering the individual’s psychological and emotional experiences, as well as their family relationships.

A review on the role of family relationships in adolescent EDs highlighted the need to refer to the adolescent’s family context to improve understanding of AN [28]. Findings from a study exploring the effects of FBT illuminated a possible mechanism of change, termed “relational containment” suggesting the importance of relational processes in recovery from AN [29]. Another study highlighted the process of evolving through treatment, for both the individual and their familial system [30]; this involved the repairing of damage through enabling others to improve their understanding of AN. The importance of attending to attachment-related issues has also been emphasised [31–36]; including the intergenerational transmission of attachment styles, emotional communication and coping styles, as well as attitudes towards eating and weight.

Whilst there is good evidence for the effectiveness of manualised ED-focussed family therapies, they do not work for everyone [37–39]. Many young people continue to experience ED-related distress following treatment, or families terminate treatment due to difficulties experienced with the approach. Accordingly, studies have attempted to identify factors that help and hinder change. Parental expressed emotion, hostility and criticism are associated with poor outcomes [40, 41], while parental warmth, increases in parental self-efficacy, and decreases in maternal critical communication and emotional over-involvement predict good outcomes [42–45]. These findings suggest that decreasing unhelpful interactions in families is important in treatment for AN. The importance of the therapeutic relationship has also been underscored, with a meta-analysis indicating that younger patients benefit from an initial focus on the alliance to build engagement [46].

In summary, expressed emotion, the therapeutic alliance, family relationships, support with understanding and managing disordered eating, attunement to the emotional and psychological experience of disordered eating, as well as developing a sense of identity outside of the ED appear to play a significant role in family therapies for AN.

### Review aims and rationale

The main limitation of family therapy treatment manuals for AN is the current lack of knowledge regarding their mechanisms of change [14]. Hence, the specific processes of change underpinning family therapies for AN remains unclear [47]. Consequently, there is a need to identify their active ingredients, and to learn who this approach works for and why [48]. This study aims to understand what helps and hinders recovery in family therapies for AN to provide insights into their processes of change. Many qualitative studies which have focussed on patient and family member perspectives of family therapies for AN have been undertaken with both adolescents and young adults [20]. The World Health Organisation’s definition of ‘young people’ covers the age range of 10 to 24 years [49]. Therefore, this review will synthesise research including adolescents and young adults aged 10 to 24 at the time of treatment, referred to henceforth as ‘young people’.

## Method

### Study design

A meta-synthesis was considered to be the most suitable approach to answer the study’s research question as it allows for the re-interpretation of meaning across a number of qualitative studies [50]. This was important given that a number of existing qualitative studies have explored the experience of treatment for AN from the perspectives of young people and families allowing for existing meta-syntheses regarding the experience of these treatments. However, no meta-syntheses have specifically explored their processes of change from these perspectives. This meta-synthesis aimed to address this gap by synthesising existing qualitative studies which have gathered data on the experience of family therapies from the perspectives of young people and families to form a new interpretation of the research, allowing for the generation of novel explanatory theory of why and how the intervention works or not.

### Search strategy

**Fig. 1:**
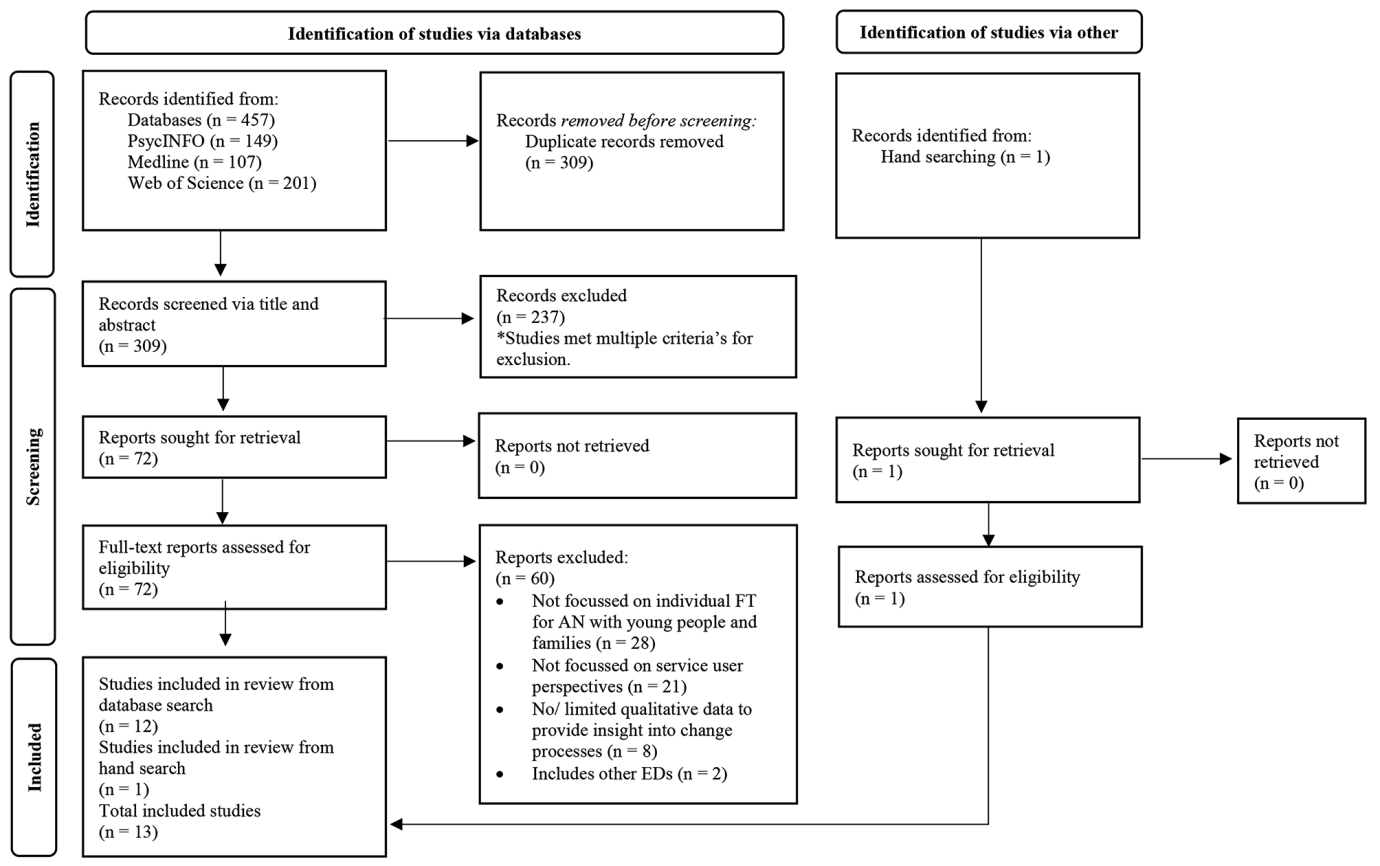
Flow of selecting and excluding studies according to the PRISMA guidelines for systematic reviews

**Fig. 2:**
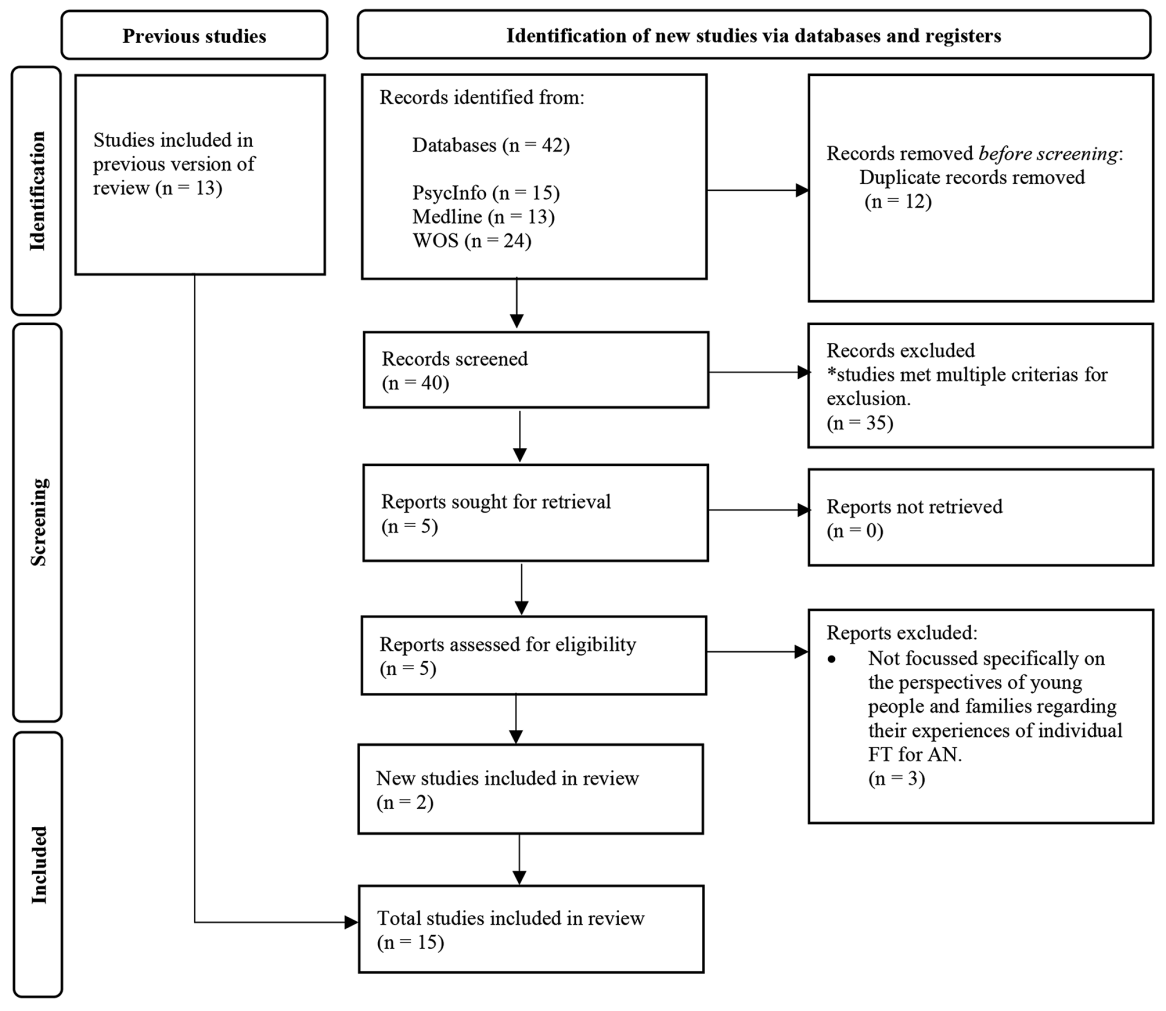
Flow of selecting and excluding studies according to the PRISMA guidelines for updated systematic reviews

This systematic review was conducted in accordance with the updated Preferred Reporting Items for Systematic Reviews and Meta-Analyses (PRISMA) guidelines [51]. The following databases were systematically searched in August 2022: PsycINFO, Medline, and Web of Science. A hand search was also conducted. The search strategy is described in Table 1 and the search results are detailed within Fig. 1. On PsycINFO and Medline, keyword searches for each concept were combined with subject heading searches using the Boolean operator ‘OR’. Web of Science does not have a search by subject heading function, therefore only a keyword search was conducted. This search was conducted again in April 2024 to account new research (see Fig. 2).

**Table 1:** Search terms and Boolean operators used to identify studies for this meta-synthesis

Key concept	Search terms
Block 1- Disorder	“Anorexi*” OR “restrictive eating disorder”
Block 2 – Therapeutic approach	“Family therap*” OR “family based treatment*” OR “family intervention” or “systemic psychotherap*” OR “systemic therap*” OR “systemic intervention*”
Block 3 – Processes of therapeutic change (phenomenon of interest)	“Family function*” OR “family relation*” OR “systemic change*” OR “family structure” OR “family dynamic*” OR “family dysfunction” OR “family role*” OR “systemic change” OR “psychotherapeutic process*” OR “therapeutic change” OR “psychotherapeutic change*” OR “behavio? r change” OR “therapeutic process” OR “strateg*” OR “interaction*” OR “pattern*” OR “support*” OR “manage*” or “attachment*” or “process* of change” OR “process*” OR “recovery process*” OR “change mechanism*” OR “change strategies” OR “change process*” OR “mechanism* of change” OR “therapeutic effect*” OR “recovery” OR “family function*” OR “help*” OR “facilitat*” OR “improve*” OR “outcome*” OR “influenc*”
Block 4 – Type of research	“Qualitative research” OR “views” OR “perspectives” OR “experience*” OR “interview*” OR “accounts”
Block 5 - Population	“Youth” OR “adolescen*” OR “teen*” OR “young people” OR “child”

### Study selection

Studies were screened at the title and abstract screening phase, and subsequently at the full text screening phase. The inclusion criteria were: (a) peer-reviewed studies published 2002 onwards following publication of the FBT manual; (b) studies employing a qualitative or mixed-method design (provided the qualitative results were derived from open-ended questions); (c) studies whose participants included young people and/ or family members who had engaged in FBT or SyFT for AN within outpatient or inpatient ED services. The exclusion criteria were: (a) studies in languages other than English; (b) and studies with mainly quantitative data, or survey data with closed questions.

### Quality appraisal

The Critical Appraisal Skills Programme tool for qualitative research was used to assess the methodological quality of papers identified [52]. The checklist has 10 criteria; each paper was given a total score out of 10. This review followed the scoring protocol used by existing ED meta-syntheses [25]. The protocol classified studies from A to C, with A denoting studies scoring 8.5 or above and carrying a low likelihood of methodological flaws; B denoting studies scoring 5 to 8, with a moderate likelihood of methodological flaws, and C indicating a score of less than five and a high likelihood of methodological flaws. Six studies scored within category A, nine studies scored within category B, and none of the studies scored within category C. The mean total score was 7.9. Scores were most commonly lost for criteria 6 and 7 (see Table 2).

**Table 2:** Quality ratings using the Critical Appraisal Skills Programme CASP tool for qualitative research

		Study number														
**CASP Criteria**	[24]	[53]	[37]	[38]	[54]	[55]	[56]	[57]	[58]	[59]	[60]	[61]	[29]	[62]	[39]	
1. Was there a clear statement of the aims of the research?	1	1	1	1	1	1	1	1	1	1	1	1	1	1		1
2. Is a qualitative methodology appropriate?	1	1	1	1	1	1	1	1	1	1	1	1	1	1		1
3. Was the research design appropriate to the aims of the research?	1	1	0.5	1	1	1	1	1	1	1	1	1	1	0.5		1
4. Was recruitment startegy appropriate to the aims?	1	0	0.5	1	1	1	1	1	1	1	1	0.5	1	1		1
5. Was data collected in a way that addressed the research issue?	1	1	0.5	1	0.5	0.5	1	0.5	1	1	0.5	1	1	0.5		1
6. Has the researcher-participant relationship been adequately considered?	0	0	0	0.5	0	0	0	0	0	0.5	0	0	0	0		0.5
7. Have ethical issues been taken into consideration?	0.5	0.5	0.5	1	0	0.5	0.5	0.5	0.5	1	0.5	0.5	0.5	0.5		0.5
8. Was the data analysis sufficiently rigorous?	1	0.5	0.5	1	0	1	0.5	1	0.5	1	1	1	1	0.5		0.5
9. Is there a clear statement of findings?	1	1	1	1	1	1	0.5	1	1	1	1	1	1	1		1
10. How valuable is the research?	1	1	1	1	1	1	0.5	1	1	1	1	1	1	1		1
Total Score	8.5	7	6.5	9.5	6.5	8.5	7	8	8	9.5	8	8	8.5	7		8.5
Total Score Category	A	B	B	A	B	A	B	B	B	A	B	B	A	B		A

*Note* Response options: 1 = Yes; 0.5 = Insufficient information to answer all criteria within item; 0 = No

### Data extraction and synthesis

This review utilised the seven-phase guidance provided by Noblit and Hare [63] for conducting a meta-synthesis which was further refined by Walsh and Downe [64]. Studies which met inclusion criteria were read in full and their results and discussion sections were extracted for analysis. Studies were then compared and translated into one another through identifying overlapping concepts (reciprocal translations) and contrasting concepts (refutational translations) across studies. Reciprocal translation was applied when concepts in one study could incorperate those of another due to their shared meaning. Reciprocal translation involved the exploration and exchanging of analogies, metaphors, themes, ideas and concepts that helped to make sense of the relationships between studies with a focus on finding analogies and explantions that best represented the whole. Refutatinal synthesis involved the identification and analysis of contradicting concepts and conclusions across studies. The concepts were subsequently synthesised to create overarching themes and subthemes (third-order concepts) whereby the identified concepts were made sense of to arrive at new interpretations. Finally, the synthesis was expressed through written form revealing more refined meanings, novel and exploratory theories.

### Reflexivity

Researcher subjectivity is inevitable within qualitative research and can be used as a valuable research tool [65]. The first author (SC) had professional experience in delivering NICE recommended treatments for adolescent AN, including manualised ED-focussed family therapies. They also had personal experience in receiving individual therapy approaches for adolescent AN, as well as non-ED focussed systemic family therapy. These experiences helped them to understand the data at an experience-near level, which strengthened the interpretative lens through which the data were read, allowing for a deeper level of analysis. However, they also paid attention to ensuring that the analysis and interpretation stayed close to the data within the primary studies by generating themes which honoured participant quotes.

In terms of researcher positionality in relation to the topic, SC reflected on their pre-conceived ideas prior to conducting this review. They acknowledged the importance of an initial focus on the restoration of eating and weight for physical health through eliciting parental management of eating behaviourals, however they also appreciated the intregral role of emotional attunement and containment throughout this process. In addition, they recognised the importance of addressing the emotional and psychological experiences underlying eating behaviours, as well as considering the young person’s family relationship context. Maintaining a reflexive journal and engaging in reflexive supervision enabled them to use their own experiences to extrapolate meanings further, whilst also broadening their perspective on the emerging themes.

## Results

### Overview of studies

Table 3 presents details of the included studies. Sample sizes ranged from *n* = 1 to *n* = 34. Six studies focussed on the experience of FBT/SyFT for AN from the perspectives of young people; four focussed on the perspectives of young people and parents; two focussed on the perspectives of young people, parents and siblings; and four focussed on parents’ perspectives alone. Data were collected from 152 young people (143 females and nine males), 189 parents and 12 siblings.

The patient age range was 11 to 23 at time of treatment, and 12 to 27 at time of data collection. The majority of patients were female (*n* = 196), 12 were male. Parent gender was discernible in twelve out of thirteen studies: 92 were mothers and 69 were fathers. Ethnicity was rarely reported. The studies were conducted in Hong Kong, China, Australia, New Zealand, Norway, United Kingdom, United States of America, Sweden and Scotland.

Nine studies collected data from participants who engaged in outpatient FBT. One collected data from participants who engaged in family sessions incorperating FBT principles within the inpatient setting. Two collected data from participants who engaged in outpatient SyFT. One collected data from participants who engaged in either outpatient FBT or SyFT and one collected data from participants who engaged in outpatient FT-AN.

**Table 3:** Characteristics of studies included in the meta-synthesis

Reference		Title	Aim	Therapeutic Approach	Sampling	Participants		Country	Data Collection	Analysis
						Description	Ethnicity			
Baudinet et al., 2024 [24]		How young people perceive change to occur in family therapy for anorexia nervosa: a qualitative study	Explore the perceived change mechanisms of FT-AN from the young person’s perspective; to shed light on possible change processes and treatment mechanisms within FT-AN.	Manualised FT-AN; outpatient	Convenience; YP were eligible if they (a) had a diagnosis of AN including atypical AN, and (b) received FT-AN at the Maudsley during the recruitment period. Potential participants were identified and invited at the point of discharge.	15 YP aged 13–18 at time of treatment and 13–19 at time of data collection. Most identitied as cisgendered female (13/15) and White. One participant identified as male and one as gender fluid. One participant identified as neuordiverse.	White British 9/15, White Other 2/15, Black British 1/15; Hispanic 1/15, Mixed 1/15.	United Kingdom	Interviews	Reflexive thematic analysis
Chan and Joyce [53]		A Feminist Family Therapy Research Study: Giving a Voice to a Girl Suffering from AN	Explore a patient’s experience of treatment by inviting her to review her family sessions.	SyFT; outpatient	Convenience; the participant was recruited within an ED service where the author worked as the family’s family therapist.	One female YP, their two siblings, mother and father. The patient was aged 21 at treatment and data collection.	Chinese	Hong Kong	Video playback and interviews	Case study analysis
Conti et al. [37]		‘Somebody Else’s Roadmap’: Lived Experience of Maudsley and FBT for Adolescent AN	Explore an experience of FBT by one family; how they negotiated their identities, roles, and alliances in a protracted phase 1.	Manualised FBT; outpatient	Purposive; the family responded to an advertisement distributed through HCP networks.	One female YP, their mother, father and male sibling who engaged in FBT for three years. The patient was aged 14 at time of data collection.	NS	Australia	Interviews	Critical discursive analysis
Conti et al. [38]		“I’m still here, but no one hears you”: a qualitative study of young women’s experiences of persistent distress post FBT for adolescent AN	Explore the experiences and identity struggles of adolescents who (1) drop out of FBT and/or (2) continue with substantive psychological distress post-treatment.	Manualised FBT; outpatient	Purposive; participants responded to advertisements on facebook or via clinicians after completing treatment.	14 female YP who engaged in FBT on average four years prior to participation for three to 24 months. Patients were aged 11–18 at treatment and 14–27 at data collection.	NS	Australia, New Zealand and UK	Interviews	Inductive thematic analysis
Krautter and Lock [54]		Is manualized family-based treatment for adolescent AN acceptable to patients? Patient satisfaction at the end of treatment	Assess the perspectives of families who completed treatment using manual-driven FBT for AN.	Manualised FBT; outpatient	Convenience; participants were invited to complete a treatment effectiveness survey at the end of treatment in an ED clinic.	34 families including 35 mothers, 31 fathers and 34 YP (32 female, two male). Treatment completion was defined as attending 80% of an average of 14 sessions over six to 12 months. Patients were aged 12–18 at data collection.	European American (*n* = 27), Asian (*n* = 3) Hispanic (*n* = 2), American Indian (*n* = 1), ‘Other’ (*n* = 1).	Northern California	Open ended survey	Phenomenological type content analysis and structural synthesis
van Langenberg, Duncan and Allen [55]		"They don’t really get heard”: A qualitative study of sibling involvement across two forms of FBT for adolescent AN.	Explore families’ experience of sibling involvement in FBT from the perspective of siblings, patients and parents.	Manualised FBT; outpatient.	Convenience; families attending a program within a specialist ED service were eligible to participate if the adolescent had at least one sibling and had completed FBT two to nine months prior.	12 siblings (10 female, one male) aged 10 to 18, 14 parents (one father, 12 mothers) and seven female patients aged 13–17 at data collection.	NS	Australia	Interviews	Thematic analysis
Lindstedt, Neander, Kjellin and Gustafsson [56]		Being me and being us - adolescents’ experiences of treatment for EDs	Investigate how YP with AN experience outpatient treatment for EDs, involving family and individual based interventions.	Flexibly adhered to FBT; outpatient	Convenience; participants were recruited in collaboration with four ED units.	15 YP (14 female, one male) were treated for AN or EDNOS restrictive over one to two years. Three received FBT, 12 engaged in a blend of FBT and individual sessions. Patients were aged 13–19 at treatment.	NS	Sweden	Interviews	Hermeneutic phenomenological approach
Joyce [57]		Patients’ perspective on family therapy for AN: A qualitative onquiry in a chinese context	Assess the perceived treatment effectiveness of family therapy from the perspective of families who have completed treatment.	SyFT; outpatient	Convenience; participants were recruited within an ED service within which the author worked as the families’ family therapist.	24 female YP and their parents treated over two and a half years. Patients were aged 14–23 at treatment.	Chinese	Hong Kong	Interviews	Content analysis
McMahon, Stoddart and Harris [58]		Rescripting—A grounded theory study of the contribution that fathers make to FBT when a young person has AN	Present a grounded theory of the contribution that fathers make to FBT when a young person has AN.	Manualised FBT; outpatient	Convenience; participants were recruited from eight CAMH services through HCPs delivering FBT.	15 fathers to a YP with AN (two male, 13 female) who had engaged in FBT. Patients were aged 11–17 at treatment.	NS	Scotland	Interviews	Classic grounded theory
Medway et al. [59]		Adolescent development in FBT for AN: Patients’ and parents’ narratives	Explore the perspectives of young people and their parents regarding the developmental impact of AN, and the role of FBT in addressing developmental challenges.	Manualised FBT; outpatient	Convenience; participants were identified from records of families who had received outpatient FBT at an ED service and had completed an adequate number of sessions of phases two and three and were weight restored.	12 young people (11 female, one male) who ceased FBT a minimum one year prior, and one of their parents (*n* = 12; 10 mothers, two fathers). Patients were aged 12–16 at onset and 16–24 at data collection.	NS	Australia	Interviews	Narrative inquiry method
Nilsen, Hage, Rø, Halvorsen and Oddli [60]		External support and personal agency - young persons’ reports on recovery after family-based inpatient treatment for AN: a qualitative descriptive study	Investigate the reflections of young persons with a lived experience of AN, and what factors they consider important for the recovery process.	Flexible adhered to FBT; Inpatient	Convenience; former inpatients were invited to participate following completion of family based inpatient treatment at an ED unit. Without adhering to manualised FBT, the guiding treatment principles were inspired by outpatient FBT. The over-arching treatment focus for the majority of participants corresponded to the first phase in outpatient FBT.	33 females and four males who were offered two family therapy sessions per week during their admission. Average length of stay was 20.8 weeks. The average time from discharge to data collection was four and a half years. Patients were aged 12–19.5 at admission and 15.8–25.3 at data collection.	NS	Norway	Interviews	Thematic analysis
Socholotiuk and Young [61]		Weight restoration in adolescent anorexia: parents’ goal-directed processes	Describe how parents participated in the weight restoration of their adolescent while engaged in FBT for AN.	Manualised FBT; outpatient	Purposive; participants responded to advertisements at local child/youth mental health centres or online. Adolescents could be in the early, middle, or late stages of weight restoration.	Three mothers and one mother-father dyad who were actively engaged in FBT. Patients were aged 13–16 at treatment.	Caucasian (*n* = 4), indigenous (*n* = 1) Parents were born in Canada (*n* = 3) and Western European countries (*n* = 2).	Canada	Video playback and systematic analysis of video recorded conversations.	Qualitative action project
Wallis et al. [29]		Relational containment: Exploring the effect of FBT for AN on familial relationships.	Investigate the process of familial relationship change for adolescents with AN and their parents who participated in FBT.	Manualised FBT; outpatient	Purposive; families who completed a 20-session protocol within an ED service at least six months before an interview with weight greater than 85% expected body weight were invited to participate.	16 female adolescents, 28 parents (15 mothers, 12 fathers) who completed a mean of 33 FBT sessions on average 12.11 months prior to data collection. Patients were 12–18 at treatment and 14–20 at data collection.	NS	Australia	Interviews	Constructionist grounded theory
Williams, Wood and Plath [62]		Parents’ experiences of family therapy for adolescent AN	Examine parents’ experiences of family therapy for adolescents with AN.	Manualised FBT (*n* = 6) or SyFT (*n* = 3); outpatient	Purposive; parents were eligible to participate if they had received or were undergoing FBT or SyFT in CAMHS, their YP was aged 12–18 years at diagnosis, their BMI was in the healthy weight range at data collection, and illness duration was less than three years prior to treatment.	Six mothers and three fathers of seven YP (six females, one male). Treatment duration ranged from 0–6 to > 36 months. Patients were 13–18 at treatment.	NS	Australia	Interviews	Interpretative phenomenological analysis
Wufong, Rhodes & Conti [39]		"We don’t really know what else we can do”: Parent experiences when adolescent distress persists after the MFT/FBT for AN.	Exploration of parents’ experiences of FBT in cases where treatment was discontinued and/or their child continued to experience psychological distress post-treatment.	Manualised FBT; outpatient	Purposive; participants responded to advertisements distributed through HCP networks.	Nine mothers and four fathers of 11 female YP who completed FBT at least one year prior. Patients were aged 12–17 at treatment.	NS	Australia	Interviews	Critical discursive analysis

† NS: Not Specified, YP: Young People, AN: Anorexia Nervosa, ED: Eating Disorder, FBT: Family Based Treatment; HCP: Healthcare Professional; CAMH: Child and Adolescent Mental Health

### Meta-synthesis findings

Six themes relating to factors that helped and hindered positive change in family therapies for AN were identified (Table 4).

**Table 4:** Overview of themes and subthemes

Theme	Subtheme
1. A holistic focus on the young person’s overall development	1a. The psychological underpinnings of AN 1b. Developing a sense of identity beyond the ED 1c. Addressing the young person in conversations about their health
2. The therapeutic relationship as a vehicle for change	2a. Young peoples’ experience of the therapist 2b. Parents’ experience of the therapist
3. The therapist’s confinement to “a script” and its impact on emotional attunement	3a. Neglect of young people’s emotional distress 3b. Neglect of family distress
4. A disempowering therapeutic context	4a. Parents “on trial” in the absence of change 4b. Rigid inflexibility versus individualised tailoring of treatment
5. Externalisation of the ED	5a. Loss of the young person’s voice and identity 5b. Separation of the problem from the young person
6. The importance of family involvement	6a. Improving understanding and management of eating behaviours 6b. Strengthening of the parent-young person relationship 6c. Changes to family functioning 6d. Complex family difficulties and relationships as a barrier to change

† AN: Anorexia Nervosa, ED: Eating Disorder

### A holistic focus on the young person’s overall development

Families considered it important to maintain a holistic focus in treatment in order to attend to the young person’s overall development including their psychological, emotional and social wellbeing, in addition to their physical health.

#### The psychological underpinnings of AN

Young people and their parents described pre-existing psychological, emotional and interpersonal difficulties which made individuation and separation from caregivers difficult [53, 59, 61]. Young people conceptualised their eating behaviours as a “coping strategy”; they used restriction as a tool for regulating emotions associated with low self-esteem, difficult life experiences, relationships, separations and transitions [60]. Some parents conveyed concern that externalising AN as “an illness” overlooked a psychological explanation for eating difficulties:

*Something causes it […] and not being able to treat what causes it, as well as the anorexia itself, trying to separate the two is a problem. And I come back to the need for a more holistic approach […] something that recognises all the complementary parts and doesn’t try and treat one in isolation of the other* [62].

Families reported how AN further impacted on young people’s development as it withdrew them from normal adolescent activities [59, 60]. Young people described feeling “stuck” and unable to relate to their peers who “continued to develop” [53, 59, 60]. Their lives had become small and narrow due to the isolating and all-consuming nature AN [24]. Feeling as though treatment attended to the young person’s emotional, social and psychological development alongside the focus on nourishment enhanced their motivation to endure distress related to making changes to their eating behaviours and weight [24, 59, 60].

#### Developing a sense of identity beyond the ED

Parents perceived the goals of weight restoration and young peoples’ development as competing and in tension [37, 55, 61, 62]; they considered it crucial to be attentive to balancing these two priorities. The requirement for parents to take responsibility for feeding the young person within phase one led to further developmental regression. Therefore, it was important that later phases of treatment focussed on supporting the development of young people’s autonomy and sense of personal identity beyond the ED [59, 60]. Parents were required to “trust” the process and their young person in order to tolerate anxiety associated with moving away from the structured routines of treatment [60, 61]:

*[…] there’s still a part of me that still really worries about it. […], but […] I need to, you know, trust that she is getting better and that she’ll start doing some of the stuff on her own* [61].

Young people who experienced more significant difficulties with their identity, anxiety and separation experienced later phases of treatment anxiety-provoking and overwhelming; this was a time of increased risk for returning to disordered eating [24, 59]. Their desire to hold on to the ED was stronger than their desire to return to normal adolescent activities. However, others were motivated to engage in changes to their eating by the prospect of engaging in valued activities and regaining an appropriate level of independence [24, 59, 60]:

*As I had more control, I felt like I was more free and more able to enjoy my social life or my schooling life or I was able to work… I was like, well this is great…How could I throw it away and go back to something like that?* [59].

Young people conveyed the importance of keeping a focus on their life outside of the ED present in treatment [24]. They emphasised the significance of engaging in meaningful aspects of life and redirecting their focus away from food and weight in order to regain a sense of personal identity beyond the ED. This included reconnecting within relationships, as well as obtaining a sense of mastery and achievement in important areas of life [24, 59, 60]:

*It’s been crucial to accomplish high school, to get a driver’s license…It feels really great to accomplish those, it’s this sense of mastering, which is very important. To feel you can live a pretty normal life, where the focus is on everything else but body and food* [60].

#### Addressing the young person in conversations about their health

Some young people spoke about experiencing a moment of clarity or insight in which they realised they were “unwell” and a subsequent realisation that something needed to come from themselves if they wanted to recover [24]. Some young people felt that being spoken to directly with factual psychoeducation within FT-AN regarding the impact of their eating behaviours on their health and functioning from trusted and credible professionals was a factor which contributed to such an awakening [24]:

*[…] he was quite harsh about it and really direct, to say that if you keep going this is what could happen. And I think that just scared me a bit but I think I needed that to scare me into recovery to begin with […]* [24].

For two young people, a shift in terms of their recovery came from trusting the dietician who joined the treatment process [24, 39]. They were able to utilise the dieticians advice and guidance to challenge their eating behaviors:

*[…] when I was faced with a situation to eat a fear food or something, I was like, Okay, remember what the dietitian said. […] And, I guess, for the eating disorder, it was like the biggest thing that was able to go against these thoughts* [24].

Hence, addressing the young person in conversations about their overall health and functioning appeared to empower and enhance their sense of personal agency in relation to their eating behaviours.

### The therapeutic relationship as a vehicle for change

When the therapeutic relationship was able to hold and contain the young person and family’s distress, it served as a vehicle for positive change.

#### Young peoples’ experiences of the therapist

On starting treatment, young people described relief coinciding with significant anxiety associated with the prospect of “letting go of control” [24, 56, 60]:

*There were two different sides within me, one saying ‘Oh God, this is great, I’m going to get help now’…At the same time, […] ‘No, now they’re going to destroy what you have achieved, and you who have come this far’* [56].

Young people emphasised the time it took for the development of trust in the therapist before they could hand over control or share their feelings [24, 56]. Feeling able to share their internal experiences was perceived as a catalyst for change [24, 56, 57]:

*An important part of the turnaround was when I invited her into my feelings. […] I started to trust her [therapist]* [56].

Hence, young people conveyed how trust and engagement needed to be prioritised from the outset [24]. They desired human connection, understanding, collaboration, compassion, and non-judgement; a therapist who was factual but who also showed genuine interest in them, rather than solely increasing their food-intake and weight [24, 53, 56, 57, 60]. When the therapeutic relationship had these qualities, young people developed confidence and trust in their therapist:

*The key to her significant improvement lies in the fact she trusted her [therapist] completely. It’s so difficult for her to trust anybody [Parent, 60].*

Young people described disengagement in the absence of a positive therapeutic relationship. Some conveyed how their disengagement was influenced by a lack of perceived understanding by their therapist, a lack of positive family relationships, or their disagreement with how weight restoration was achieved [56, 59].

#### Parents’ experiences of the therapist

A positive therapeutic relationship was important in providing parents with emotional containment [29, 57, 62]. Parents desired a clinician who took an active role in aiding parental understanding and support of the young person, as well as family communication [29, 57, 58, 62]:

*She [therapist] knew how to teach us to deal with my daughter’s problem and that’s most memorable* [57].

The therapist was experienced positively when they had a neutral quality and were compassionate, collaborative, helpful, flexible and placed emphasis on building trust [29, 37, 39, 57, 58, 62]. Parents wanted to feel connected with their therapist, have their concerns contextualised and validated, and their circumstances and values respected [29, 37, 39, 57, 58, 62]:

*We have built up mutual trust. There’s genuine concern between her [therapist] and us. She didn’t treat us as if it were her job or profession* [57].

Moreover, parents wanted to improve their knowledge of AN in order to feel confident in their capacities to help [58, 61]. A lack of adequate information and guidance given by the therapist contributed to a lack of containment experienced within the therapeutic relationship in FBT [58]:

*[…] It seemed to be pushing all the onus on correction and enforcement on to my wife and I, and nothing coming from the clinicians, no support for us in our battle.* [58].

Some parents perceived the therapist as cold, harsh, scrutinising, condescending and detached in FBT [29, 37, 39, 57, 58, 62]. One parent made a distinction between a positive experience of SyFT versus a negative experience of FBT, describing the SyFT therapist as coming “alongside” the family, as opposed to the FBT therapist acting as a “removed expert” [62].

### The therapist’s confinement to a “script” and its impact on emotional attunement

Families conveyed how positive change was hindered by a confined focus on food intake and weight in FBT.

#### Neglect of young people’s emotional distress

Young people frequently described FBT as an isolating process that ignored “what was going on inside” wherein their parents and therapist did not understand how distressing increasing their eating and weight was [37, 38, 54, 59]:

*[…] they just solely focused on my physical health, they didn’t really take on much consideration as to my mental health and how much of a toll everything was taking on me* [37].

Young people desired support with managing the psychological experience of AN and its associated difficulties rather than solely focussing on increasing their food-intake and weight in FBT [57]. Areas of importance included motivation, emotions, thoughts, relationship difficulties, self-esteem, body image and identity issues [37, 38, 54, 58–60]:

*Because they never really addressed the underlying problems, it was all so much harder than it probably should have been, because I was still battling with the thoughts and the guilt* [56].

Some young people felt that important conversations (i.e., the impact of their parent’s attitudes to eating and weight) were not sufficiently acknowledged in FBT [38]:

*We opened old wounds and then they never really got closed I never got to just express how I was really feeling, which is probably why I was so angry, because it was all, like building up inside* [38].

Some parents emphasised how FBT assumes that weight restoration leads to cognitive change [37, 38]; they expressed concerns that the psychological underpinnings of AN and its associated difficulties were unaddressed by the “FBT script” [37–39, 54, 55, 59–61]:

*The focus seems to be all on the food aspects […] the food is the end product of the whole problem, what’s going on underneath?* [39].

When young people were not restoring weight in FBT, parents were concerned by the continued confined focus on food intake and weight as the young person became increasingly distressed and conflicts worsened [39]. They questioned whether FBT was doing more harm than good; for some, this led to treatment termination [37, 38, 56]. For those who engaged in FT-AN, some young people felt that the early stages of treatment were very parent focussed which inhibited their ability to share their internal experiences and resulted in them struggling to engage due to the experience of distress. However, some young people felt that the middle and later phases of FT-AN did attend to their psychological and emotional needs which had a positive impact on their recovery [24]:

*[…] early treatment was very much nutrition focused. It was kind of let’s just get her fed enough. And then middle treatment was kind of unpacking lots of fears. […] just things like thoughts around food […]. And then end of treatment for me was kind of looking at what might have caused it, how you can prevent it* [24].

#### Neglect of family distress

Parents often described how being redirected to the “FBT script” caused distancing in the therapeutic relationship and reduced confidence about therapy [29, 37, 58, 62]. Many families conveyed a lack of space for the exploration of familial distress and conflict, which for some contributed to family estrangement and exacerbation of distress in FBT [37, 62]:

*FBT ruined a previously strong relationship and caused my parents and siblings their own psychological unease and detriment. This contributed to a loss of myself and my identity and resulted in further destructive behaviours [Young person, 37].*

Parents conveyed how the task of “refeeding” as the solution obscured how demanding, distressing and dilemmatic their experience was in FBT. They considered it important to feel resourced emotionally, practically and personally to create capacity for the task of weight restoration; parental management of emotion was significant in this respect [61]. They felt overwhelmed, exhausted, isolated and let down by the therapist when their challenges were not recognised or supported in FBT [29, 37, 39, 58]:

*[…] it sounds easy in principle, […] when you actually do it at home it’s not that easy when a teenager’s screaming at the top of her lungs […] you feel a little thrown to the wolves […] once you close the door of your family home that’s it; you’re on your own* [62].

Parents engaged in an internal search for answers regarding the cause for their young person’s eating difficulties to provide them with a sense of coherence and to guide day-to-day decision making in FBT [39, 58, 61]. Parents acknowledged the therapists’ attempts to relieve them of guilt and blame with FBT’s agnostic aetiological stance, however described the experience of persistent guilt, self-blame and inadequacy [29, 54, 58, 62]:

*[…] it felt like, well as a parent, what have we done wrong?* [39].

Parental co-regulation was a strategy for the management of difficult emotions [37, 61], as were internal processes such as practicing acceptance, managing expectations and finding meaning in adversity [61]. However, practical challenges to parents’ capacity such as the lack of time and financial resources posed significant dilemmas for single parents engaged in FBT [61, 62]:

*I can’t take any time off work, I don’t have any money, I don’t know how I’m going to help her!* [61].

### A disempowering therapeutic context

Families conveyed how the experience of disempowerment in treatment was hindering to positive change.

#### Parents “on trial” in the absence of change

It was important to parents that treatment monitored progress in the overall health and wellbeing of their young person [61]. Attending to markers of progress was a powerful motivator that instilled hope, or revealed what was not working [61]. However, some parents described FBT as a “ruthless”, “dogmatic” approach in which they felt “put on trial” [37, 39]. These parents reported to receive blaming, punitive responses from the therapist when there was insufficient weight restoration in FBT. Such interactions exacerbated parental guilt and self-blame, causing parents to feel exposed and vulnerable [37, 39]:

*I have never been so challenged in my life as a mother. I felt wretched. It’s such a fundamental thing to feed and protect your child and anorexia has already challenged that […] then to, on a weekly basis, be in a context (emotional tone to voice) where your failings are on show and also on show to your children* [39].

Parents reported that these interactions implicitly conveyed how they were failing in their role as parents and at FBT [37, 39], particularly when the therapist endorsed the assumption that “the Maudsley works”:

* I felt like she was on trial […]. Hayley’s failure to put on weight was Margaret’s failure to feed her enough ultimately* [39].

#### Rigid inflexibility versus individualised tailoring of treatment

Some families reported to feel that FBT was “rigid”, “prescribed” and “inflexible” [37, 39]. Families who engaged in FBT or FT-AN would have liked more separated sessions in addition to conjoint sessions throughout treatment in order that parents’ and young peoples’ individual emotional needs could be met [24, 37, 54, 61]:

*[…] there were other times where it was just really awkward talking about my deepest, darkest anorexia secrets, I definitely was able to open up more when it was just one on one with my psychologist, about actual behaviours, because you don’t want to admit those kind of things to your parents [Young person, 46].*

Families who engaged in FBT reported a lack of tailoring to preferences regarding session format and content, treatment duration, ending, and opportunity to have a follow up [37, 54, 58]:

*The rigidity and inflexibility [,,,] was such a shame […] because […] the heavy lifting was done, which was the trust […]. The lack of progress or our frustration or anything to just flex the approach and to lean on the trust that had been built. Well, I think we’d have gone to the edge of the earth with her you know until it became so rigid, dogmatic […] [Parent, 36].*

Some families reported that time-limited FBT was not sufficient to support a full recovery [39, 54, 56]. Young people wanted the therapist to be attuned to their needs and to make a collaborative decision rather than ending treatment on reaching a “target weight” in FBT [56]. The therapists’ perceived assumption that they were psychologically recovered on reaching a healthy weight evoked feelings of anger and abandonment:

*OK, now you have reached normal weight - so now you are well again. The simple fact that I had put on weight meant that everything was fine, and that was all there was to it. I felt like…I’m not prepared to walk out weighing like this, if you leave me, I will start losing weight again* [56].

### Externalisation of the eating disorder

Externalisation could help and hinder positive change depending on how it was used and its effects on the young person and their relationships.

#### Loss of the young person’s voice and identity

Some parents described how young people were spoken about rather than with as a result of externalisation, which contributed to a perceived loss of the young person’s voice and identity in FBT [37, 38]:

*[…] I accept that when she was very sick, […] we talk in these beautiful terms of ‘It was the eating disorder and not Hayley in the room’ […] but effectively Hayley was still in the room but she was treated as though she wasn’t* [37].

Some young people who engaged in FBT did not find it helpful for others to perceive of them as being under the influence of an external entity because they felt unseen and unheard [37, 38]. Moreover, externalising conversations that highlighted family member burden due to AN were particularly difficult for some as they evoked guilt and shame. Instead, young people wanted their voice to be actively sought and to be given ownership over session content, rather than feel “talked about” and instructed what to do in an impersonalised manner in FBT [37, 56]:

*[…] the fact that you had an eating disorder meant they were dismissive of anything you say, they believed anything you say was completely motivated by the eating disorder […]. I was very distressed by that because I thought I’m still me, I’m still here, I can recognise that I have anxiety and unhelpful thoughts but I can still communicate as a person* [38].

In contrast, a collaborative involvement in treatment fostered greater security and safety in the young person’s relationships with their therapist and family [24, 37, 53, 56, 60]. Feeling seen as a person beyond the ED helped to build young people’s sense of self, rather than reinforce an illness identity through the exclusion of their voice.

#### Separation of the problem from the young person

At the start of treatment, conceptualising AN as an ‘unwanted temporary illness’ enabled parents to channel difficult emotions into “fighting” AN [37–39, 54].

*There was Sally and there was the eating disorder and once you separate them you realise that she is still there and we’re fighting the eating disorder and she is too. […] it wasn’t just her doing this to herself* [39].

Some young people conveyed how important it was for their family to realise that EDs are illnesses rather than ‘difficult behaviour’ [24]. The illness metaphor enabled some young people to feel seen and understood “as a person” by their therapist and family [24]. Feeling related to as a person experiencing an illness was validating and containing, rather than dismissive and invalidating:

*My [therapist] was really good at explaining to my parents and my family: ‘Actually this isn’t a choice. You know, this isn’t something [young person’s name] woke up one day and decide to do to herself, this is an illness. I’m going to treat it like an illness* [24].

However, it was important that externalising conversations validated the young person’s lived experience. For instance, Venn diagrams acknowledging overlaps between the identity of the young person and that of AN, as well as engaging in chair work to speak to AN were described as meeting young peoples’ emotional and psychological needs [38]:

*[…] they’d be like, what would your eating disorder say to this? Now sit in this chair and it’d be like, what would you say to this? […] that was helpful, but they just didn’t do it enough. Like, it was just so much about food but they needed to care about my* feelings [38].

Young people emphasised the significance of transitioning from “denial” to “realisation” through externalising conversations which elicited the young person’s voice and facilitated reflection on their personal values and life aspirations [37, 60].

*Ask yourself, why, why do you do this? What do you want to get out of your life? What are your true dreams? What is your greatest wish?* [60].

Young people acknowledged the importance, yet difficulty, of becoming aware of AN’s negative effects and its function in their lives [24, 60]:

*It’s important to realize and see more clearly the negative influences the ED has, because it is, after all, a way of handling difficulties or mastering life. […] It’s not easy to be attentive to the negative consequences the ED will have* [60].

Young people stressed their anxieties and resistance to making changes to their eating. However, they tolerated difficult feelings in the service of their commitment to a preferred identity underpinned by their personal values [24, 60]:

*I haven’t thought much about having kids. Still, I think it is important to stay in treatment, because I want to be able to take good care of my kids, which is a huge motivation for me* [60].

### The importance of family involvement

Interventions aimed at improving parental understanding and management of AN, as well as family functioning were supportive of positive change.

#### Improving parental understanding and management of AN

Parents entered treatment feeling powerless, not knowing how to help their young person [62]. For some, the structure and guidance on commencing FBT provided direction which instilled hope [29, 37, 39, 56, 61, 62]:

*I felt totally out of control. […] So, having a plan just made me feel like I had something secure to-to work on, to work with, to trust in* [39].

One mother conveyed how FBT gave her permission and resources to challenge their young person’s eating behaviour:

*Treatment gave me tools and framework and a structure and permission I suppose* [29].

Some young people experienced relief, safety and security when control over their eating was taken away in FBT/FT-AN [24, 29, 37, 38]. Most young people acknowledged that they would not have restored weight without parental support with challenging the ED in the process of adhering to their eating plan [24, 29, 37, 38, 60]:

*[…] I don’t think I would have been able to gain that weight and get to the medically stable point if it had sort of been all up to me. […] I still definitely needed my family support because […] even if it was unintentionally, I would’ve just slipped back […]* [37].

Young people conveyed the perceived helpfulness of psychoeducation and guidance from the therapist and treatment team which enabled their parents to understand and respond to their experiences more effectively [24, 29, 37, 38, 60]. Hence, promoting change at a systemic level was integral to their recovery:

*[…] as much as it was about teaching me the correct thoughts, and the correct patterns, […] how to get better. It was also teaching my parents what the right things were to say and what the right behaviours were, and what actually they had done that was maybe not so healthy* [24].

A consistent, firm approach to managing eating behaviours was containing to young people in the sense that the parental boundary allowed no escape route for the ED [24]. Parents reflected on the potential of AN to undermine the marital relationship and the importance of parental unification and partnership which involved co-parent coordination and negotiation [39, 58, 60–62]. Thus, it was helpful when treatment scaffolded the sharing of roles and responsibilities between co-parents to ease parental burden as well as to ensure that both parents were engaged in changes conducive to young peoples’ recovery [61, 62]:

*We were a joint force. I think if you can work together really quickly it helps because an eating disorder can get around one of you, but if it knows that dad’s there backing up everything that mum says and vice-versa […]* [39].

Parents who were not supported in treatment by their co-parent would have liked to have shared parental responsibility and were concerned about their co-parent’s lack of understanding for their young person’s difficulties [61, 62]. In the context of parental separation, it was important that both parents pulled together to preserve the young person’s sense of unified family support [62]. This necessitated parental acceptance of disparate parenting philosophies and weight-restoration strategies [62]:

*At first it was like, […] “Hello? Uh, that’s not happening”. And then, you know, it’s like, “Actually, we need to do this for [our daughter]. Darn. Okay”. So suck it up, bury those emotions* [62].

#### Strengthening of the parent-young person relationship

Whilst some parents felt that treatment disrupted the parent-young person relationship in FBT [37, 38], others felt it helped them to maintain a connection with their young person. Some parents described personal changes which served to strengthen the parent-young person relationship, for instance becoming firmer with boundaries, more resilient and emotionally attuned [29, 39, 58, 61, 62]:

*I was one of these people who didn’t show my emotions much, very tough orientated, very much the job at hand…once I understood what anorexia was like for my son, it helped me change. Now I’ve let my guard down and let people in. […] It’s [AN] made me a better person and a better parent…* [62].

Young people acknowledged how difficult it was for their parents to understand AN, however their efforts to do so made them feel worthy of care [24, 38, 56, 61, 62]. In the context of relationships in which young people felt seen, heard, understood and cared for, they were more accepting of the structures put in place to support them with eating [24, 29, 37, 38, 56, 60]. Thus, young people found it helpful when the therapist mobilised emotionally containing relationships [24, 38, 56, 59]. They conveyed the importance of parental support in helping them to cope with ED-related cognitions and emotions which felt too overwhelming and distressing for them to deal with on their own [24]:

*[…] there’s a point where the eating disorder’s voice is stronger than anything else in you. And so you are not physically able to fight that yourself. You need help. You need other people to, like, to help fight that voice until you’re capable of doing that yourself* [24].

Parental capacity to take non-coercive control over the young person’s eating appeared to be influenced by parents’ capacity to tolerate the young person’s distress [29]. When treatment contained parental anxiety and increased parental confidence, parents had greater capacity to provide emotional containment to the young person [24, 29, 61]. Consequently, over time young people experienced greater security, safety and trust in the parent-young person relationship and were able to replace control with trust in others [24, 29, 37, 56, 59, 61]:

*I felt the need to be in control but […] I sort of had to put my trust in to them that they were going to take care of me* [29].

Increased relational security in the parent-young person relationship was depicted by young people as learning to understand each other, reduced parental criticism, and increased parental provision of emotional support and reassurance [24, 29].

*They tried to be as understanding as they could be [parents] […] it helped because for the first time I didn’t actually shun them, I went to them…it’s so weird cause for the first time in my life I let myself depend on someone else* [29].

Parents perceived the quality of connection between themselves and their young person as a tangible marker of positive progress in treatment [29, 61]. These relational changes had a positive impact on young peoples’ attachment system and sense of selves. Young people described reduced self-criticism, improved confidence, greater self-acceptance, increased capacity to trust others and in turn, in their selves [29, 61]. Parents described reduced secretiveness, increased resilience and emotional expressiveness in their young person [29, 61].

#### Changes to family functioning

Many families entered treatment with strained family relationships. Some young people felt they were a “burden” and “a stranger” within their family [29]:

*We weren’t being like a family, a bit separated, like I was separated from everyone else in the family* [29].

In the context of family sessions which permitted space for therapeutic focus on relationships, positive changes to family functioning were reported, including improved communication, cohesion and problem-solving skills; increased honesty, openness and closeness; mutual understanding and trust; emotional awareness and tolerance; reduced conflict, criticism and blame; and increased capability and compatibility [24, 29, 39, 54–58, 61, 62]. Having a safe, contained space for difficult conversations helped to reduce emotional disconnection:

*They […] guided us through […] very hard conversations. Hard as in about feelings, about guilt, about perceptions, and about the effects it had on us […] I think as a family we’ve grown a lot closer […] [Parent, 38].*

Families often described more space to focus on the family system within SyFT, FT-AN and flexibly adhered to FBT as oppose to strictly adhered to FBT [24, 53, 57, 62]. They valued the therapist’s directiveness on what needed to change, and their appreciation for each person’s feelings and views [24, 57]. These processes helped young people to share their feelings [24, 57]:

*What helped most is to talk about my feelings without any reservation. She helped everyone to speak out, including those who didn’t talk much [Young person, 60].*

Some young people felt that through the process of conversations which supported the family to engage with one another on an emotional level, they began to mentalise the experience of their parents and siblings which had a positive impact on their commitment to recovery [24, 57]:

*I remember there was a little bit of guilt for what I was putting my parents through…and in a strange way, I kind of think that guilt was a motivator as well, because I was kind of like, I cannot keep doing this to my parents. They cannot live like this forever* [24].

Some fathers became more involved in their young person’s life practically and emotionally through their involvement in treatment [29, 53, 58]. One young person described how her father’s involvement helped to reduce the disconnection between them:

*[…] I can see that my father also tries to show concern for me. That feeling has been lost for years. He loves me and really wants to help me* [53].

Siblings struggled to understand AN and wanted to be included in treatment [55, 56]. When siblings were not included, sibling-relationship ruptures were often left unrepaired causing young people to experience loss, sadness and guilt. Families reported positive aspects of sibling involvement, including increased understanding and unified support, increased closeness in the sibling relationship, reduced sibling worry, greater transparency in the family system and opportunity to clarify appropriate family roles [55]:

*I think it made it so [sibling] understood […] what was going on. So it wasn’t like she was kept in the dark and we were trying to avoid talking about it to her. So she was a bit more aware of what was going on and she didn’t feel left out of it. She knew what not to say […]* [55].

#### Complex family difficulties and relationships as a barrier to change

It was important that treatment addressed parental belief systems which served to maintain young peoples’ disordered relationships with eating and weight [38, 61]. One mother viewed slimness to confer protective factors for their young person and thus experienced challenge and unease during the process of refeeding. In order for treatment to facilitate weight restoration, the mother’s own beliefs in relation to eating and weight needed to be explored and addressed as an ongoing process:

*The thinner you are, the more beautiful you are, the easier the world is for you. I truly…believe that. […] You want your kid to be….to fit in, […] and obviously that’s easier if you look a certain way […]. What if they’re wrong? What if they make her gain too much weight, and then…and then she feels like she’s…too heavy* [61].

Young people did not perceive parental involvement to be helpful when they did not have trusting relationships with their parents, or when they had family difficulties which were not openly discussed in treatment [29, 56, 59]. They expressed uncertainty about what they were able to share due to concerns about disclosing their parents’ own difficulties, or revealing family conflict:

*My parents have alcohol problems […] I preferred going on my own. […] the fact is I never told anyone at the eating disorder unit. […] I was so ashamed […] I was so dead scared of what might happen if they learnt about it* [56].

These young people explained how there were difficulties which felt out of their control that they needed help with before letting go of control over their eating [59]; they described poorer relationships with parents, family conflict and high expressed emotions at home. Unresolved family difficulties and conflicts led to unrepaired ruptures and disconnection in their family relationships following treatment. Some families for whom relational change did not eventuate were experiencing grief, loss and trauma [29]. For example, one young person explained that previous abuse in the family impacted on her ability to trust her parents [30]:

*[…] I didn’t trust my parents […] it would just have been so strange to have my mum sitting there. Then I would not have dared to be honest in the same way* [56].

## Discussion

This meta-synthesis explored the processes of change in family therapies for AN, elucidating factors that help and hinder recovery from the perspectives of young people and their families. The majority of studies explored young person and family member experiences of FBT, or SyFT; only one study explored the experience of FT-AN and this was from the perspectives of young people alone. The narratives depict several therapeutic processes which appear integral to facilitating positive change including psychological formulation, identity development beyond AN, the therapeutic relationship, emotional attunement, family involvement and family empowerment. These findings are discussed in relation to existing research, theory and their clinical implications.

### Psychological formulation

Restriction provided young people with a sense of control over psychological and emotional difficulties, however it also resulted in the loss of agency and stalled development. Whilst FBT often failed to explore and address the psychological underpinnings of AN, parental re-feeding was often successful at supporting physical recovery and enabling young people to return to developmentally appropriate activities. This finding suggests an experience of change that aligns with proposed mechanisms of change within ED-focussed family therapy models [13, 14]. FBT and FT-AN aim to get the adolescent ‘developmentally back on track’ through the process of re-feeding which allows them to return to normal adolescent activities. Some young people experienced their return to valued activities reinforcing of their sense of personal agency which had a positive impact on their recovery. Research demonstrates how shifts in motivation for change occur in parallel with shifts in values and self-definitions through a process of identity renegotiation [66]. The findings alongside existing research underscore the significance of relinquishing control, self-discovery, and the development of a new identity in recovery from an ED which provides a false sense of security [67–69]. Accordingly, empowering young people to identify and return to developmentally appropriate activities that bring personal meaning and identity may help young people to connect with what is important to them and thereby support them in working towards and maintaining recovery. Ensuring that life outside of the ED is brought into treatment may help to aid such processes in order to facilitate positive change.

The findings underscore the importance of maintaining a holistic focus on the young person’s overall development in treatment, attending to their psychological, emotional, social and physical health to support positive change. Families often conveyed how FBT viewed the young person’s eating behaviours in isolation of their overall development which resulted in a perceived lack of understanding for the cause and maintenance of eating difficulties, and other associated difficulties. Therefore, the experience of families suggests that seeking to understand the young person’s eating difficulties within the context of their overall development is an important change mechanism in family therapies for AN. This finding does not align with what the FBT model proposes as a change mechanism. Rather, FBT takes an agnostic approach; it does not focus on exploring causes of AN and instead aims to engage the family in bringing about early behavioural change, assuming that an understanding of the cause does not necessarily translate to symptom improvement [17].

The finding that FBT’s agnostic aetiological stance does not alleviate parental guilt and blame is consistent with existing research. Carers are often perplexed by ANs cause, place blame on themselves and question their parenting [70, 71]. Hence, they place value on the opportunity to improve their understanding [72]. Caregiver cognitive appraisals relating to their understanding of AN are important because they have a direct impact on caregiver self-efficacy, the caregiving experience and their responses to the individual with AN [70]. This meta-synthesis suggests that some parents feel frustrated by FBT’s agnostic aetiological stance and continue to seek a causal explanation to make sense of their young person’s eating difficulties. Gorrell & Le Grange [73] state that the non-blaming foundation that ED-focussed family therapy models rest upon should not be confused within inattention to, and avoidance of exploring why struggles arise and persist. The authors state that treatment stagnation may be better understood if caregivers are invited to more directly speculate about how their own temperament, family attachment history, and distress tolerance may be getting in the way of positive change. 

ED-focussed family therapy models place emphasis on externalising the ED as an ‘illness’ or external force to facilitate positive change. However, the findings suggest that the illness metaphor may have contributed to a neglect of psychological formulation for the young person’s eating difficulties in FBT. In turn, families experienced concern regarding FBT’s efficacy in meeting young peoples’ psychological and emotional needs. Some FBT clinicians experience the illness metaphor dilemmatic as it does not fit with their psycho-social understanding of EDs, however they continue to use it as recommended in the manual [74]. Baudinet, Simic and Eisler [18] underscore the importance psychological formulation in ED-focussed family therapy; proposing that it is through the formulation that treatment can be adjusted to target maintenance factors specific to individual families and that barriers to progress can be identified and overcome. Moreover, formulating collaboratively with a family can create a shared narrative about the problem and support the development of therapeutic alliance [75].

It has been proposed that AN arises from emotional processing difficulties resulting in a ‘lost sense of emotional self’ which ‘self-perpetuates’ and ‘relentlessly deepens’ [76]. Psychological factors including obsessive-compulsive personality traits, perfectionism, extreme need for self-control, cognitive rigidity, experiential avoidance, positive beliefs about the value/function of AN, and responses from close others play a significant role in its maintenance [77–79]. Developing a psychological formulation would help therapists to develop a holistic understanding of the young person within their family context, prioritise which issues to focus on, and select interventions to address not only the physical symptoms of AN, but also its psychological underpinnings and maintaining factors within family therapies for AN.

Positively, ED-focussed family therapy has been updated within FT-AN to consider the development of an evolving systemic formulation to be an important framework for thinking about change with the family throughout treatment. According to the FT-AN manual, the development of a systemic formulation includes consideration of (1) the nature of the problems that the young person and family are struggling with; (2) reorganisation of the family around the illness; (3) problem narratives, beliefs and cognitions; (4) emotions and feelings that may be connected to the illness; (5) mapping significant patterns; (6) strengths, resources and resilience factors. Further, the FT-AN model suggests that whilst eating and weight are likely to be the primary therapeutic focus at least initially, an important context for thinking about the process of change is to broaden the focus to other areas that are seen to contribute or perpetuate the ED, or are in other ways important to the family [14]. Therefore, the change mechanisms proposed by FT-AN may better aligned with what families perceive as being important factors in facilitating change through its attendance to young peoples’ eating behaviour within the context of their overall development and family environment. However, given that only one study exploring the experience of FT-AN was included within this review, it is difficult to ascertain whether FT-AN is more able than FBT to meet the needs of families and to thereby support positive change through its updated emphasis on formulation.

### The therapeutic relationship

The finding that the therapeutic relationship is experienced as a crucial mechanism of change aligns with what ED-focussed family therapy models propose as being an important mechanism of change [13, 14]. The therapist’s knowledge, flexibility and willingness to come alongside them were conveyed by families as being particularly significant in supporting positive change. In contrast, negative experiences of the therapist acting as a ‘removed expert’ were viewed as a hinderance to positive change. In FBT the therapist takes up a non-authoritarian collaborative therapeutic stance [17]. They adopt the position of being an expert on EDs, while they view parents as being the expert in their young person and family in the hope of empowering parents to bring about recovery. The findings indicate that families valued the therapist taking up the position of expert in EDs as it enabled them to feel held and supported in managing the young person’s eating difficulties. However, they indicate that parents can experience a lack of containment in FBT when they perceive a lack of direct guidance from the therapist. This finding suggests that therapists’ attempts to build self-reliance by guiding and not giving explicit instructions may be experienced as challenging for parents when they are struggling to bring about change in their young person’s eating and weight and are in need of more direct guidance.

ED-focussed family therapy has been updated within FT-AN to initially emphasise engagement with the family and young person by creating a containing and secure base for treatment in which a shared sense of purpose is developed [14]. Accordingly, the FT-AN model acknowledges that the provision of expert advice can build dependency on the therapist but sees this as an appropriate aspect of the early stage of treatment with potential pitfalls that therapists should be aware of as treatment progresses. Therefore, it is possible that positive change may be helped by the provision of more direct guidance in the face of parental struggle with bringing about change in family therapies for AN. In doing so, families may experience the therapeutic relationship as a containing and secure base in which they can learn new skills and knowledges to manage their young person’s eating difficulties.

The findings support reviews which point to the significance of building trusting therapeutic relationships with young people and families [1, 27]. They also support studies which demonstrate significant associations between therapeutic alliance, treatment retention and outcome for individuals with AN [80–83]. Entering a therapeutic relationship required young people to relinquish control and place trust in others which necessitated the tolerance of heightened distress. A common theme raised by families is a concern about the lack of therapeutic alliance in treatment for AN [1, 84]. The findings in combination with existing research suggest that individuals want to feel seen and treated as a ‘whole person’, within a ‘real’ relationship with a therapist who is attuned to their emotional and psychological experiences rather than neglectful of their distress in the pursuit of weight restoration [1, 23, 25–27, 85, 86]. Attention to building and maintaining a strong therapeutic alliance with young people and their families by attending to and repairing ruptures, seeking to accurately understand their experiences, clarifying expectations and mutually agreeing treatment goals appear crucial to facilitating positive change.

Negative experiences of the therapeutic relationship were more common within manualised FBT than in SyFT, FT-AN, or flexibly adhered to FBT. Manual-based treatments have been criticised for contributing to ‘bland’, ‘rule governed’, and ‘emotionally detached’ therapy [87]. Clinical situations can differ from the tightly controlled conditions of a clinical research study in which evidence for a manual-based treatment is developed as clinicians are often required to adapt their practice to meet individual needs [88]. Therefore, it has been argued that while fidelity is a crucial component of successful evidence-based psychotherapy, flexible implementation allowing for deviation from the manual to individualise treatment is necessary [89]. The findings alongside existing research stress a need for greater flexibility in the delivery of manualised FBT. Nevertheless, despite being a manual-based treatment, FT-AN appeared to be experienced more positively by young people within this review. Thus, FT-AN may be superior to FBT in its ability to establish and maintain positive therapeutic relationships through its emphasis on engagement with all family members from the outset and use of formulation to individualise treatment.

### Emotional attunement

The finding that positive change was hindered by the experience of confinement in focus on food intake and weight does not align with change mechanisms proposed by ED-focussed family therapy models. FBT in particular takes a pragmatic approach maintaining a ‘laser like focus’ on symptom reduction to elicit change [17]. Hence, emotional and psychological distress which is perceived by the therapist as secondary to the ED is not visited until ‘the crisis is over’ and the young person is physically well. In doing so, the intention is to remain focussed on eating behaviours to support the restoration of physical health which is thought to lead to the alleviation of emotional and psychological distress related to the ED. Concerningly, the narratives of young people and families who engaged in FBT depict an experience of treatment which was at times neglectful of their distress as a consequence of this focus.

The updated ED-focussed family therapy model, FT-AN, views the containment and validation of family anxiety as crucial in supporting positive change (Eisler et al., 2016). The model proposes that therapists need to be aware of how a decrease in anorexic cognitions does not necessarily run in parallel with weight gain. Further, that for some young people anorexic cognitions and other difficulties (e.g., anxiety, ocd and depression) may become more difficult as weight increases, causing distress for both the individual and family. FT-AN suggests that for young people in this situation, offering coinciding individual therapy could be beneficial within phase three. However, young people who engaged in FT-AN described a need for treatment to attend more to their internal experiences within the earlier phases of treatment as they were more parent-focussed. Therefore, positive change in family therapies for AN may be supported by increasing their attention to the emotional experiences of the young person in the earlier phases of treatment. A focus on emotional attunement alongside the focus on the restoration of physical health through increasing food intake and weight may support young people’s engagement throughout the entirety of treatment through aiding the development of a therapeutic relationship in which the young person experiences a sense of emotional containment.

Many young people reported to feel that what they were experiencing internally “did not matter” within FBT. Some healthcare professionals (HCPs) treating AN feel that the biomedical model supports them to define target symptoms and goals for recovery, however families perceive the model to place too much focus on the physical, ignoring psychological distress [27]. This meta-synthesis alongside existing research illustrates how families seek a holistic individually-adapted treatment that is flexible, and considers the psychology of the young person’s eating behaviour, as well as their family environment [1, 23, 25, 27]. Some FBT clinicians assume that weight restoration is the primary agent of cognitive symptom relief through the alleviation of cognitive rigidity resulting from starvation [90]. However, this meta-synthesis alongside existing research suggests that the psychological experience of AN can persist beyond weight restoration [90]. Thus, therapists should be cognisant of the messages conveyed to families through holding this assumption as they may risk invalidating their lived experiences and hindering the alliance. The findings suggests that whilst ED-focussed family therapies can support weight restoration, many young people experience ongoing difficulties in their relationship to eating and weight which they would like support for within individual therapy, alongside and/or following completion of family therapy for AN.

Some HCPs working within ED services express a lack of knowledge about EDs, and perceive limitations in their capacity to help [27, 91–94]. ED groups are considered difficult to treat because they present with physical and psychological risks, as well as ambivalence [94]. For clinicians, the FBT “script” can be experienced as a relief in terms of managing their own anxiety [91]. Yet, the manual can also be experienced as constraining, morally dilemmatic and burdensome when there is no positive change; nonetheless, clinicians fear scrutiny if they do not practice with strict adherence [91]. The narratives within this meta-synthesis reveal the impact of this dilemma on therapeutic change. Whilst therapists’ drift from evidence-based practice is suggested to reduce treatment effectiveness [95], adherence to the FBT manual is not associated with outcome as determined by weight [96]. This meta-synthesis suggests that strict adherence to the FBT manual may reduce therapist attunement, which in turn may hinder positive change by negatively impacting on the alliance.

Research has shown that family therapy focussing on intra-familial dynamics rather than ED symptoms improves treatment effectiveness in severe adolescent AN, indicating the broader impact of family therapy beyond the effects of parental management of eating behaviours [97]. Research comparing FBT with a generic systemic manual (SyFT) found that those who engaged in FBT restored weight more promptly in treatment and spent less time in hospital than those who engaged in SyFT, whereas individuals with greater obsessive-compulsive comorbidity restored more weight over the course of treatment in SyFT than in FBT [98]. Therefore, there is likely to be overlap between SyFT and FBT in terms of guidance on managing eating behaviours, however therapists delivering SyFT may have greater capacity to be attuned and responsive to individual needs as a result of feeling less constrained by the FBT manual.

Some researchers have suggested that evidence-based ED-focussed family therapy models require more flexible, normative, less guilt-inducing, diversified, elective and integrative practices [99]. If FBT and SyFT achieve equivalent outcomes, and SyFT may be more effective at addressing comorbidity and creating a family environment conducive to positive change, there may be scope to expand focus beyond food-intake and weight within FBT. With greater flexibility and integration of models, therapists could attend to the psychological and emotional **e**xperience of AN, as well as the individual’s relational context which may provide families with a greater sense of containment. It has been suggested that FBT can be enhanced by integrating Emotion-Focussed Family Therapy (EFFT) with the aims of working with parents’ own emotions and supporting parents to equip the young person with emotion regulation skills [100]. Families highlighted dissatisfaction with FBT for neglecting their emotional experiences. Hence, the findings support the integration of EFFT to aid therapeutic change in family therapies for AN.

Nonetheless, whilst the current findings are discussed in relation to potential adjustments to the FBT model, these reflections are responding to an assumed better experience. It is important to note that we do not currently have knowledge about their relationship to outcome without further research. It is possible that therapists may find it harder to learn or implement the model if there was more flexibility and scope for augmentation. This may result in poorer treatment that is less precise and potentially less effective. Hence, this is currently an unknown risk. Importantly, as only one study focussed on the experience of FT-AN within the current review, it is difficult to ascertain whether this updated model of ED-focussed family therapy is experienced as less neglectful of young people and families’ distress. However, young people generally spoke more positively about FT-AN as oppose to FBT. Therefore, it is possible that FT-AN may be better able to hold and contain families’ distress.

### Empowering families

The finding that positive change can be hindered by the family’s experience of a disempowering therapeutic context does not align with how ED-focussed family therapy models aim to bring about therapeutic change. ED-focussed family therapy models assume their ability to effect change through empowering parents to take on the challenging task of ensuring that the young person eats an appropriate amount of food and thus, interrupting ED behaviours [13, 14]. Some parents perceived a harsh response from the therapist in the absence of sufficient weight restoration during FBT. In the context of non-successful outcomes, FBT clinicians often attribute causality to parents or family systems factors [89]; this assumption aligns with parents’ narratives within this meta-synthesis. Parental self-blame has been captured within this study, as well as in existing research [71]. Concerningly, interactions which inadvertently disempower parents may hinder positive change by negatively impacting on the therapeutic relationship and reducing parental self-efficacy.

The perceived experience of harshness from the therapist may reflect FBT’s emphasis on ‘intensifying the crisis’ to effect behavioural change as soon as possible. However, the ED-focussed family therapy model has since evolved with FT-AN now more focussed on ensuring that parents feel contained and supported themselves to ensure effective problem solving, emotional warmth, validation and calm persistence in the face of their young peron’s distress [19]. Given the absence of data regarding parents’ experiences of FT-AN within the current review, it is not possible to make conclusions about their experience of this approach. However, it is possible that FT-AN may be experienced more positively than FBT by parents through its focus on containing and supporting parents.

Furthermore, the findings suggest that positive change can be hindered by the experience of rigidity and inflexibility within FBT. Lock and Nicholls [101] maintain that whilst FBT initially targets weight restoration, it also addresses temperament/personality traits, emotional processing, cognitive content and process, social communication and relationships, comorbidity and family factors. However, some families did not feel that they were given sufficient opportunity to address such factors. Lavender [84] asserts that it is possible to achieve a full recovery using FBT and suggests that allowing the young person more individual time can support with alliance building, with phase three being extended to focus on areas of difficulty that made the young person vulnerable to AN. This meta-synthesis suggests that therapeutic change may be helped through the provision of greater flexibility in terms of treatment duration, content and format in family therapies for AN.

With little data regarding families’ experiences of FT-AN, it is difficult to establish whether this updated approach is more able to create an empowering therapeutic context than FBT. It is possible that FT-AN is experienced as less rigid and inflexible given that it has been updated to specifically emphasise the engagement of all family members from the outset of treatment, including the young person, and the use of formulation to ensure that treatment is individually tailored [18, 19]. Whilst the narratives of young people who engaged in FT-AN depicted a treatment which was confined in its focus on food intake and weight, some young people expressed a need for more individual time throughout the course of treatment to support them with managing emotional and psychological distress related to the ED. Therefore, it is possible that providing young people with more targeted individual time alongside family sessions throughout the entirety of treatment may help to support positive change in family therapies for AN.

### Externalisation

The finding that the conceptualisation of AN as an ‘unwanted temporary illness’ was mobilising of parental compassion and support is consistent with change mechanisms proposed by ED-focussed family therapy models. ED-focussed family therapy manuals suggest that viewing AN as an illness or external force gives new meaning to AN’s physical, psychological and behavioural effects [13, 14]. In doing so, externalisation is thought to elicit change by encouraging parents to separate the illness from the adolescent, and to thereby task parents with ‘battling’ the ED, not their young person [17]. Whilst this meta-synthesis reveals narratives which align with this theory of change, they also provide further insight by demonstrating how externalising language can cause relational disconnection when young people feel unseen and unheard because all of their views and behaviours are ascribed to AN. FBT clinicians report that externalisation can improve family functioning, communication, and reduce family conflict; however, they also highlight the importance of listening skills and attunement to identify how and when to time externalisation, including recognition of when a family may not benefit [74]. Thus, whilst externalisation appears to be an important change mechanism in family therapies for AN, it is likely to be most effective in the context of therapeutic skill and knowledge and should be used carefully, sensitively and collaboratively to avoid its potential pitfalls as listed within the FT-AN manual [14].

The findings are consistent with existing research in that they underscore a prevalent negative experience of ED service-users which is feeling treated as an ‘illness’ [1, 25, 102]. It has been suggested that implicit value judgement carried by ‘illness’ may lead to negative interactions between service-users and healthcare professionals resulting in epistemic injustice [103]. This meta-synthesis alongside existing research demonstrates that individuals value a non-judgemental, respectful and supportive therapist [38]. Therefore, therapists should be careful that their use of externalisation enables individuals to feel recognised and respected as a person beyond AN. The findings of this review are consistent with novel research exploring service-user experiences of externalisation in therapies for AN [104]. Together, the narratives depict how externalising practices are most helpful when led by the individual using their own experience-near language. Further, that they are least helpful when externalising language does not permit the individual to feel seen as a person beyond AN. Individuals seek to develop a psychologically informed understanding of their experiences of AN, as well as to strengthen an alternative narrative identity in line with their personal values [104]. Hence, therapists should be cognisant of the emotional effects of language used to externalise AN and ensure that such practices do not occlude the young person and family’s opportunity to develop a psychologically informed understanding of their experiences of AN. Moreover, whilst AN and its impact on the individual and family may be the dominant story early on in treatment, therapists could endeavour to co-develop other narrative identity stories available to the family, those that are less problem saturated. In doing so, the therapist may engage the family in conversations which serve to thicken the more subjugated stories of their lives and empower them in relation to their experience of AN.

Conversations in which the therapist actively involved the young person to reflect on their values and aspirations alongside exploration of the function and effects of their eating behaviours helped to separate AN from the young person’s identity. Motivation, insight and subjective meaning of AN are valuable tools to manage resistance [105]. Successful treatment facilitates the development of an identity that is separate to and broader than that defined AN [106]. Hence, an important part of motivating young people to engage in changes to their eating behaviour may be to engage them in externalising conversations which place emphasis on identity development. Language which empowers individuals in relation to their eating difficulties and which helps individuals to feel seen, heard, and validated as a person beyond AN is supportive of recovery [104]. Accordingly, practicing externalisation with adherence to the principles of narrative therapy as suggested by Michael White, the founder of this practice [107, 108], may positively impact on therapeutic change in family therapies for AN.

### Family involvement

ED-focussed family therapy models assume their ability to bring about change through their emphasis on parental involvement, viewing parents as the primary agents of change in the recovery process. The narratives within this review align with this proposed change mechanism and suggest that parental involvement is crucial for physical recovery. This finding also aligns with the reports of FBT clinicians that parental input is crucial for weight-based symptom remission [89]. Parents experience fear related to their involvement in the recovery process [109]; maternal fear predicts lower self-efficacy, as well as more accommodating and enabling behaviours. This meta-synthesis highlights the importance of supporting parents to feel equipped in helping their young person to manage their eating behaviours. Caring for someone with an ED is challenging and family members often experience high levels of burden and distress [110]. Gorrell & Le Grange [73] underscore the importance of not overlooking the pravers of youth with EDs, and presents a compelling call to action for the ED field to better support parents who navigate treatment. This meta-synthesis supports an existing quantitative review which emphasises the important role of parental behaviour in facilitating a good treatment outcome; specifically, parental empowerment to elicit early weight gain and parental criticism have been shown to be influential in treatment for AN [111]. Therapeutic efforts to hold and contain parental distress by ensuring that parents feel adequately equipped to help their young person manage the ED, any comorbidity, and adolescence as a developmental stage, as well as attuning to parents’ emotional experiences may in help to increase parental self-efficacy and reduce parental criticism.

Parents experienced containment within treatment when they felt supported by their therapist [29]. In turn, they were able to provide their young person with containment through becoming emotionally attuned, understanding and validating of their experiences. This finding suggests that relational processes are needed at multiple levels to contain and hold. Containment denotes the relational ability to ‘hold’ the emotion that the other person needs held and to create a felt sense of safety in the relational space [112, 113]. Attunement refers to the parent’s awareness of and responsiveness to the young person’s emotions and needs, and ability to stay present with them even when that feels difficult [114]. The effect is that the young person feels understood, seen and felt with by the parent; this attuning process grows the capacity for felt safety in relationships [114]. The findings are consistent with research which has shown that increases in parental self-efficacy throughout treatment predict reduced ED, depression and anxiety symptoms [115]. It is possible that when parents feel ‘held’ by the therapist, they feel more confident in their ability to support their young person, which in turn enables the young person to feel more ‘held’ within their family system.

The narratives depict how positive relational changes within the parent-young person relationship were internalised by young people, and in turn positively impacted on young peoples’ emotional and psychological wellbeing. Hence, this meta-synthesis supports quantitative research which demonstrates that family therapy for AN can support positive changes to the parent-young person relationship, which in turn has a positive impact on outcome [44]. Positive affective family relationships are crucial with respect to adolescent eating behaviour and emotional adaptive psychological functioning [28]. The findings alongside existing research underscore the importance of nurturing secure parent-adolescent attachment relationships to support positive change.

This meta-synthesis uncovered narratives of intergenerational patterns in emotional expression and interpersonal relating that impacted the parent-young person relationship [62], as well as the intergenerational transmission of attitudes towards eating and weight [38]. Exploring transgenerational family relationship patterns and their influence in the development of coping strategies and identity within individuals experiencing AN can determine areas for development in the family, as well as treatment strategies [116].This meta-synthesis underscores the importance of family involvement and the value of therapeutic space to focus on family relationships. However, there was greater opportunity to enhance family unity within SyFT and FT-AN as oppose to FBT. Thus, the findings suggest that families may benefit from greater systemic family focus within FBT.

Relatedly, the findings underscore the importance of scaffolding parental unification in the treatment of AN. Research suggests that the presence of co-parental conflict is associated with lower adolescent BMI and to more dysfunctional family functioning [117]. Hence, attending to the co-parent relationship may help to support positive change. Assessing and modifying family functioning early in treatment may be of particular benefit to young people who have more complex family difficulties. Adolescents experiencing AN present with more severe psychological profiles within families that display more severe dysfunctional profiles [118]. Research indicates a bi-directional relationship between family dysfunction and AN, with difficult dynamics and unhelpful cycles becoming entrenched [119]. Families of adolescents with AN often report interpersonal boundary problems, low conflict tolerance, and low general satisfaction within the family system [120]. Adolescents who report more positive views of their family at the start of treatment have better outcomes in FBT [121]; hence, it is important to assess family functioning in order that adolescent reported impairment can promote the delivery of family therapy for AN with greater relational focus from the outset. This modification may foster relational containment within the young person’s family system, creating a conducive environment for reducing disordered eating.

Attachment-based family therapy can serve as a beneficial augmentation through its focus on repairing factors that impact relational security and maintain AN (i.e., parental criticism and low warmth, family conflict and adolescent affect intolerance) [36]. Research on attachment and narrative theory in SyFT indicates the common presence of extreme separation anxiety, unresolved trauma and loss, as well as conflict avoidance and difficulties discussing relationships and feelings consistent with transgenerational experiences of insecure/avoidant attachments in young people with EDs and their families [34, 122]. These authors suggest that helping families to explore their experiences from a secure base can foster ability to reflect on their relationships, externalise the past, and relate to one another in emotionally different ways. Hence, an attachment-informed relational focus in family therapies for AN may support positive change for families experiencing more complex relational difficulties.

Lastly, this meta-synthesis illustrates how weight restoration takes place within relational systems that are shaped by sociocultural and other contextual variables. Recognising factors which under-resource parents such as the influence of financial strain and time constraints is important, particularly for those who are unsupported by a co-parent. Research suggests that socioeconomic factors do not predict outcome in FBT [123], however this meta-synthesis reveals how some families are faced with significant challenge when they are required to reduce their income to fulfil the role required of them within FBT. This may become more problematic in the global cost-of-living crisis [124]. To support positive change, future research and ED services could give consideration to adaptations that may support families who struggle to meet the demands of ED-focussed family therapy models in the face of socio-economic pressures.

### Strengths, limitations and future research

A limitation of this review is that data analysis was completed by only the first author (SC). However, they engaged in reflexive discussion in relation to the data throughout the process of theme generation within supervision. The themes and illustrative quotes were subsequently reviewed by both supervisors (MP and LS) once written up and their feedback was incorporated to further refine and/ or expand the theme. These processes ensured that whilst SC led on the translation and synthesis of concepts across studies, the over-arching themes reflect multiple perspectives.

Another limitation is that the majority of studies explored the experience of FBT or SyFT within the outpatient setting. Only one study explored the experience of FT-AN within the outpatient setting and this was from the perspectives of young people. Thus, no studies explored the experience of FT-AN from the perspectives of parents or siblings. Therefore, it is not possible to make conclusions about family member experiences of FT-AN and this is an important area for future research. Additionally, only one study explored the experience of treatment guided by FBT principles within the inpatient setting. Future research could explore their processes of change more specifically as the structure of treatment within the inpatient setting will be different to that within the outpatient setting.

A further limitation is that the systematic search strategy did not include the term ‘young adults’. This is because ED-focussed family therapy is the first line recommended treatment for adolescent AN [10], whereas young adults may be offered individual CBT-ED or MANTRA as first line treatments for AN [125]. Therefore, the review initially set out to include studies gathering the perspectives of adolescents and their family members. However, following having completed the searches, it was evident that many studies included both adolescents and young adults, using the term ‘young people’ to describe their sample. Consequently, given the lack of existing research on change processes within family therapies for AN, the decision was made to be inclusive by including studies that collected data from both adolescents, young adults and their family members. Nonetheless, the United Nations define ‘youth’ as persons between the ages of 14 and 24 years [126], the NHS uses the term ‘young people’ to classify individuals aged 16 to 24 years [127], and the World Health Organisation defines ‘young persons’ as those aged 10 to 24 years [128]. Therefore, it is possible that this limitation was somewhat mitigated through inclusion of the search terms ‘youth’ and ‘young people’.

Furthermore, a large proportion of patients were female and ethnic background was rarely reported. This information may have provided insight into differences in perceptions of therapeutic change across ethnicities and genders. Both gender and ethnicity influence the experience of AN [129, 130]. Therefore, whilst it is difficult to make conclusions about the ethnicity of participants represented within this review, the experience of those who identify as male or non-binary are under-represented. Reporting ethnicity in future studies and increasing the diversity of samples may enhance our understanding of under-researched groups.

Moreover, attempts to narrow the search strategy to ensure the study’s feasibility within a restricted timeframe resulted in the exclusion of unpublished studies which may have contributed to the loss of information [131]. However, unpublished studies may be of lower methodological quality [132]. Alongside the consideration of conceptual quality which is an important component of qualitative research [133], the methodological quality of included studies was critically appraised using the CASP tool. Nevertheless, studies often lacked sufficient detail to answer all items on this tool, in which case the item was scored zero. Studies rarely engaged in reflexivity in relation to their position as a researcher which is an important consideration in qualitative research [134]. Future research should aim to report how the researcher has examined their own influence on all parts of the research process.

Lastly, the findings illuminate the need to explore the role of the therapist in establishing and maintaining a strong therapeutic alliance in family therapies for AN as this was a key driver of therapeutic change. They also indicate a need for clinicians, ED services and researchers to consider how ED-focussed family therapy models could more effectively meet young people and families’ emotional and psychological needs. This review’s suggested changes to family therapies for AN could be evaluated using measures of therapeutic alliance, family functioning, ED symptoms and common comorbidities.

## Conclusions

This review highlights the importance of facilitating positive relational changes within the family system, exploring, understanding and addressing the psychological and emotional experiences of AN, supporting young people to develop a sense of personal identity beyond the ED, and creating an empowering and supportive therapeutic context in which young people and their families experience containment within the therapeutic relationship. Young people and families perceived positive change to be hindered by inflexibility in the treatment approach, a lack of positive therapeutic alliance and attunement to their emotional experiences, counter-effects of externalisation such as the exclusion of the individual’s voice, and a narrow focus on food-intake and weight. The findings can be utilised by ED services to consider how they may adapt to the needs of young people and their families. For example, by broadening the focus of treatment beyond restoring food intake and weight to attending to the young person’s emotional needs and facilitating relational containment within their family system throughout the entirety of treatment.

## Data Availability

No datasets were generated or analysed during the current study.

## Abbreviations

UK: United Kingdom
AN: Anorexia nervosa
ED: Eating disorder
FBT: Family based treatment for Anorexia nervosa
FT-AN: Family therapy for Anorexia nervosa

## References

[1] Sibeoni J, Orri M, Valentin M, Podlipski MA, Colin S, Pradere J, Revah-Levy A. Metasynthesis of the views about treatment of anorexia nervosa in adolescents: perspectives of adolescents, parents, and professionals. PLoS ONE. 2017;12(1):e0169493. 10.1371/journal.pone.0169493. 28056106 10.1371/journal.pone.0169493 PMC5215824

[2] van Hoeken D, Hoek HW. Review of the burden of eating disorders: mortality, disability, costs, quality of life, and family burden. Curr Opin Psychiatry. 2020;33(6):521. 10.1097/YCO.000000000000064. 32796186 10.1097/YCO.000000000000064 PMC7575017

[3] Whitney J, Eisler I. Theoretical and empirical models around caring for someone with an eating disorder: the reorganization of family life and inter-personal maintenance factors. J Mental Health. 2005;14(6):575–85. 10.1080/09638230500347889. 10.1080/09638230500347889

[4] Lock JD, Fitzpatrick KK. Anorexia nervosa. BMJ Clin Evid 2009; 1011. https://pubmed.ncbi.nlm.nih.gov/19445758/. PMC2907776 19445758

[5] Zipfel S, Giel KE, Bulik CM, Hay P, Schmidt U. Anorexia nervosa: aetiology, assessment, and treatment. Lancet Psychiatry. 2015;2(12):1099–111. https://doi.org/10.•1016/S2215-0366(15)00356-9. 26514083 10.1016/S2215-0366(15)00356-9

[6] Culbert KM, Racine SE, Klump KL. Research Review: what we have learned about the causes of eating disorders–a synthesis of sociocultural, psychological, and biological research. J Child Psychol Psychiatry. 2015;56(11):1141–64. 10.1111/jcpp.12441. 26095891 10.1111/jcpp.12441

[7] Hinney A, Volckmar AL. Genetics of eating disorders. Curr Psychiatry Rep. 2013;15:1–9. 10.1007/s11920-013-0423-y. 10.1007/s11920-013-0423-y 24202964

[8] Lavender JM, Wonderlich SA, Engel SG, Gordon KH, Kaye WH, Mitchell JE. Dimensions of emotion dysregulation in anorexia nervosa and bulimia nervosa: a conceptual review of the empirical literature. Clin Psychol Rev. 2015;40:111–22. 10.1016/j.cpr.2015.05.010. 26112760 10.1016/j.cpr.2015.05.010 PMC4537813

[9] Treasure J, Willmott D, Ambwani S, Cardi V, Clark Bryan D, Rowlands K, Schmidt U. Cognitive interpersonal model for anorexia nervosa revisited: the perpetuating factors that contribute to the development of the severe and enduring illness. J Clin Med. 2020;9(3):630. 10.3390/jcm9030630. 32120847 10.3390/jcm9030630 PMC7141127

[10] National Institute of Care Excellence. Anorexia nervosa: treatment for children and young people. Information for the public. Eating disorders: recognition and treatment. Guidance NICE. 2017. https://www.nice.org.uk/guidance/ng69/ifp/chapter/Anorexia-nervosa-treatment-for-children-and-young-people.

[11] Watson WH. Family systems. In: Elsevier eBooks. 2012. pp. 184–93. 10.1016/b978-0-12-375000-6.00169-5.

[12] Grange DL, Lock J, Loeb KL, Nicholls D. Academy for eating disorders position paper: the role of the family in eating disorders. Int J Eat Disord. 2009;43(1):1–5. 10.1002/eat.20751. 10.1002/eat.20751 19728372

[13] Lock J, Le Grange D. Treatment manual for anorexia nervosa: a family-based approach. Guilford; 2015.

[14] Eisler I, Simic M, Blessitt E, Dodge L. Team. Maudsley Service Manual for Child and Adolescent Eating disorders (revised). Child and adolescent eating disorders Service. London: South London & Maudsley NHS Foundation Trust; 2016. http://www.national.slam.nhs.uk/services/camhs/camhs-eatingdisorders/resources/.

[15] Eisler I, Wallis A, Dodge E. What’s new is old and what’s old is new: The origins and evolution of eating disorders family therapy. Family Therapy for Adolescent Eating and Weight disorders: New Applications. Taylor and Francis Inc; 2015. pp. 6–42. 10.4324/9781315882444.

[16] Gorrell S, Simic M, Grange DL. Toward the integration of Family therapy and Family-Based Treatment for Eating Disorders. In: Springer eBooks. 2023. pp. 1–17. 10.1007/978-3-030-97416-9_59-1.

[17] Rienecke RD, Grange DL. The five tenets of family-based treatment for adolescent eating disorders. J Eat Disorders. 2022;10(1). 10.1186/s40337-022-00585-y. 10.1186/s40337-022-00585-y PMC9066936 35505444

[18] Baudinet J, Simic M, Eisler I. Formulation in eating disorder focused family therapy: why, when and how? J Eat Disord. 2021a;9(1):97. 10.1186/s40337-021-00451-3. 34376258 10.1186/s40337-021-00451-3 PMC8353776

[19] Baudinet J, Simic M, Eisler I. From treatment models to manuals: Maudsley single and multi-family therapy for adolescent eating disorders. In: Mariotti M, Saba G, Stratton P, editors. Systemic approaches to manuals. 1st ed. Cham: Springer; 2021b. pp. 349–72. 10.1007/978-3-030-73640-8.

[20] Fisher CA, Skocic S, Rutherford KA, Hetrick SE. Family therapy approaches for anorexia nervosa: a cochrane review. BJPsych Adv. 2020;26(3):130. 10.1002/14651858.CD004780.pub3. 10.1002/14651858.CD004780.pub3 PMC6517149 30320438

[21] Pote H, Stratton P, Cottrell D, Shapiro DA, Boston P. Systemic family therapy can be manualized: research process and findings. J Family Therapy. 2003;25(3):236–62. 10.1111/1467-6427.00247. 10.1111/1467-6427.00247

[22] Cottrell D, Boston P, Practitioner, Review. The effectiveness of systemic family therapy for children and adolescents. J Child Psychol Psychiatry. 2002;43(5):573–86. 10.1111/1469-7610.00047. 12120854 10.1111/1469-7610.00047

[23] Medway M, Rhodes P. Young people’s experience of family therapy for anorexia nervosa: a qualitative meta-synthesis. Adv Eat Disorders. 2016;4(2):189–207. 10.1080/21662630.2016.1164609. 10.1080/21662630.2016.1164609

[24] Baudinet J, Eisler I, Konstantellou A, Simic M, Schmidt U. How young people perceive change to occur in family therapy for anorexia nervosa: a qualitative study. J Eat Disorders. 2024;12(1). 10.1186/s40337-024-00971-8. 10.1186/s40337-024-00971-8 PMC10804743 38254187

[25] Babb C, Jones CRG, Fox JRE. Investigating service users’ perspectives of eating disorder services: a meta-synthesis. Clin Psychol Psychother. 2022;29(4):1276–96. 10.1002/cpp.2723. 35141970 10.1002/cpp.2723 PMC9546143

[26] Bezance J, Holliday J. Adolescents with Anorexia Nervosa have their Say: a review of qualitative studies on treatment and recovery from Anorexia Nervosa. Eur Eat Disorders Rev. 2013;21(5):352–60. 10.1002/erv.2239. 10.1002/erv.2239 23765431

[27] Gustafsson SA, Stenström K, Olofsson H, Pettersson A, Ramsay KW. Experiences of eating disorders from the perspectives of patients, family members and health care professionals: a meta-review of qualitative evidence syntheses. J Eat Disorders. 2021;9(1). 10.1186/s40337-021-00507-4. 10.1186/s40337-021-00507-4 PMC8642844 34863276

[28] Erriu M, Cimino S, Cerniglia L. The role of Family relationships in Eating disorders in adolescents: a narrative review. Behav Sci. 2020;10(4):71. 10.3390/bs10040071. 32252365 10.3390/bs10040071 PMC7226005

[29] Wallis A, Rhodes P, Dawson L, Miskovic-Wheatley J, Madden S, Touyz S. Relational containment: exploring the effect of family-based treatment for anorexia on familial relationships. J Eat Disorders. 2017;5(1). 10.1186/s40337-017-0156-0. 10.1186/s40337-017-0156-0 PMC5532769 28770090

[30] Coopey E, Johnson G. Exploring the experience of young people receiving treatment for an eating disorder: family therapy for anorexia nervosa and multi-family therapy in an inpatient setting. J Eat Disorders. 2022;10(1). 10.1186/s40337-022-00609-7. 10.1186/s40337-022-00609-7 PMC9277598 35831883

[31] Dallos R. Attachment narrative therapy: integrating ideas from narrative and attachment theory in systemic family therapy with eating disorders. J Family Therapy. 2004;26(1):40–65. 10.1111/j.1467-6427.2004.00266.x. 10.1111/j.1467-6427.2004.00266.x

[32] Dallos R, Vetere A. Systemic therapy and attachment narratives: applications in a range of clinical settings. Routledge; 2021.

[33] Gander M, Sevecke K, Buchheim A. Eating disorders in adolescence: attachment issues from a developmental perspective. Front Psychol. 2015;6:1136. 10.3389/fpsyg.2015.01136. 26321974 10.3389/fpsyg.2015.01136 PMC4530258

[34] O’Shaughnessy R, Dallos R. Attachment research and eating disorders: a review of the literature. Clin Child Psychol Psychiatry. 2009;14(4):559–74. 10.1177/1359104509339082. 19759074 10.1177/1359104509339082

[35] Sherkow SP, Kamens SR, Megyes M, Loewenthal L. A Clinical Study of the Intergenerational Transmission of Eating Disorders from Mothers to Daughters. The Psychoanalytic Study of the Child. 2009 1;64(1):153–89. 10.1080/00797308.2009.11800819. 10.1080/00797308.2009.11800819 20578438

[36] Wagner I, Diamond G, Levy S, Russon J, Litster R. Attachment-based family therapy as an adjunct to Family‐Based treatment for adolescent anorexia nervosa. Australian New Z J Family Therapy. 2016;37(2):207–27. 10.1002/anzf. 10.1002/anzf

[37] Conti J, Calder J, Cibralic S, Rhodes P, Meade T, Hewson D. Somebody else’s Roadmap’: lived experience of Maudsley and family-based therapy for adolescent anorexia nervosa. Australian New Z J Family Therapy. 2017;38(3):405–29. 10.1002/anzf.1229. 10.1002/anzf.1229

[38] Conti J, Joyce C, Natoli S, Skeoch K, Hay P. I’m still here, but no one hears you: a qualitative study of young women’s experiences of persistent distress post family-based treatment for adolescent anorexia nervosa. J Eat Disorders. 2021;9(1). 10.1186/s40337-021-00496-4. 10.1186/s40337-021-00496-4 PMC8588656 34772464

[39] Wufong E, Rhodes P, Conti J. We don’t really know what else we can do: parent experiences when adolescent distress persists after the Maudsley and family-based therapies for anorexia nervosa. J Eat Disorders. 2019;7(1). 10.1186/s40337-019-0235-5. 10.1186/s40337-019-0235-5 PMC6373134 30805186

[40] Eisler I, Simic M, Russell GFM, Dare C. A randomised controlled treatment trial of two forms of family therapy in adolescent anorexia nervosa: a five-year follow‐up. J Child Psychol Psychiatry. 2007;48(6):552–60. 10.1111/j.1469-7610.2007.01726.x. 17537071 10.1111/j.1469-7610.2007.01726.x

[41] Rienecke RD, Accurso EC, Lock J, Grange DL, Expressed, Emotion. Family Functioning, and treatment outcome for adolescents with Anorexia Nervosa. Eur Eat Disorders Rev. 2015;24(1):43–51. 10.1002/erv.2389. 10.1002/erv.2389 PMC4962527 26201083

[42] Byrne CE, Accurso EC, Arnow KD, Lock J, Grange DL. An exploratory examination of patient and parental self-efficacy as predictors of weight gain in adolescents with anorexia nervosa. Int J Eat Disord. 2015;48(7):883–8. 10.1002/eat.22376. 25808269 10.1002/eat.22376 PMC4845658

[43] Grange DL, Lock J, Agras WS, Moye A, Bryson SW, Jo B, et al. Moderators and mediators of remission in family-based treatment and adolescent focused therapy for anorexia nervosa. Behav Res Ther. 2012;50(2):85–92. 10.1016/j.brat.2011.11.003. 22172564 10.1016/j.brat.2011.11.003 PMC3260378

[44] Moskovich AA, Timko CA, Honeycutt LK, Zucker N, Merwin RM. Change in expressed emotion and treatment outcome in adolescent anorexia nervosa. Eat Disord. 2016;25(1):80–91. 10.1080/10640266.2016.1255111. 27869569 10.1080/10640266.2016.1255111 PMC6361377

[45] Sadeh-Sharvit S, Arnow KD, Osipov L, Lock J, Jo B, Pajarito S, et al. Are parental self‐efficacy and family flexibility mediators of treatment for anorexia nervosa? Int J Eat Disord. 2018;51(3):275–80. 10.1002/eat.22826. 29314160 10.1002/eat.22826 PMC6756483

[46] Graves TA, Tabri N, Thompson-Brenner H, Franko DL, Eddy KT, Bourion-Bédès S, et al. A meta‐analysis of the relation between therapeutic alliance and treatment outcome in eating disorders. Int J Eat Disord. 2017;50(4):323–40. 10.1002/eat.22672. 28152196 10.1002/eat.22672

[47] Blessitt E, Voulgari S, Eisler I. Family therapy for adolescent anorexia nervosa. Curr Opin Psychiatry. 2015;28(6):455–60. 10.1097/yco.0000000000000193. 26382158 10.1097/yco.0000000000000193

[48] Jewell T, Blessitt E, Stewart C, Simic M, Eisler I. Family therapy for child and adolescent eating disorders: a critical review. Fam Process. 2016;55(3):577–94. 10.1111/famp.12242. 27543373 10.1111/famp.12242

[49] World Health Organization: WHO. Adolescent and young adult health [Internet]. 2023. https://www.who.int/news-room/fact-sheets/detail/adolescents-health-risks-and-solutions.

[50] Atkins S, Lewin S, Smith H, Engel ME, Fretheim A, Volmink J. Conducting a meta-ethnography of qualitative literature: lessons learnt. BMC Med Res Methodol. 2008;8(1). 10.1186/1471-2288-8-21. 10.1186/1471-2288-8-21 PMC2374791 18416812

[51] Page MJ, McKenzie JE, Bossuyt PM, Boutron I, Hoffmann T, Mulrow CD, et al. The PRISMA 2020 statement: an updated guideline for reporting systematic reviews. BM. 2021;71. 10.1136/bmj.n71. 10.1136/bmj.n71 PMC8005924 33782057

[52] Critical Appraisal Skills Programme. CASP Qualitative Studies Checklist. 2018. https://casp-uk.net/checklists/casp-qualitative-studies-checklist-fillable.pdf.

[53] Chan ZCY, Joyce LC. A Feminist family therapy research study. J Feminist Family Therapy. 2006;17(2):41–64. 10.1300/j086v17n02_03. 10.1300/j086v17n02_03

[54] Krautter TH, Lock J. Is manualized family-based treatment for adolescent anorexia nervosa acceptable to patients? Patient satisfaction at the end of treatment. J Family Therapy. 2004;26(1):66–82. 10.1111/j.1467-6427.2004.00267.x. 10.1111/j.1467-6427.2004.00267.x

[55] Van Langenberg T, Duncan RE, Allen JS, Sawyer SΜ, Grange DL, Hughes EK. They don’t really get heard: a qualitative study of sibling involvement across two forms of family-based treatment for adolescent anorexia nervosa. Eat Disord. 2018;26(4):373–87. 10.1080/10640266.2018.1453632. 29683775 10.1080/10640266.2018.1453632

[56] Lindstedt K, Neander K, Kjellin L, Gustafsson SA. Being me and being us - adolescents’ experiences of treatment for eating disorders. J Eat Disorders. 2015;3(1). 10.1186/s40337-015-0051-5. 10.1186/s40337-015-0051-5 PMC4381673 25834734

[57] Joyce LC. Patients’ perspective on family therapy for anorexia nervosa: a qualitative inquiry in a Chinese context. Australian New Z J Family Therapy. 2008;29(1):10–6. 10.1375/anft.29.1.10. 10.1375/anft.29.1.10

[58] McMahon K, Stoddart K, Harris F. Rescripting—A grounded theory study of the contribution that fathers make to family-based treatment when a young person has anorexia nervosa. J Clin Nurs. 2021;31(11–12):1598–611. 10.1111/jocn.16013. 34448286 10.1111/jocn.16013

[59] Medway M, Rhodes P, Dawson L, Miskovic-Wheatley J, Wallis A, Madden S. Adolescent development in family-based treatment for anorexia nervosa: patients’ and parents’ narratives. Clin Child Psychol Psychiatry. 2018;24(1):129–43. 10.1177/1359104518792293. 30080102 10.1177/1359104518792293

[60] Nilsen JV, Hage TW, Rø Ø, Halvorsen I, Oddli HW. External support and personal agency - young persons’ reports on recovery after family-based inpatient treatment for anorexia nervosa: a qualitative descriptive study. J Eat Disorders. 2020;8(1). 10.1186/s40337-020-00293-5. 10.1186/s40337-020-00293-5 PMC7197126 32391150

[61] Socholotiuk KD, Young RA. Weight restoration in adolescent anorexia: parents’ goal-directed processes. J Eat Disorders. 2022;10(1):190. 10.1186/s40337-022-00676-w. 10.1186/s40337-022-00676-w PMC9730571 36476504

[62] Williams L, Wood C, Plath D. Parents’ experiences of family therapy for adolescent anorexia nervosa. Australian Social Work. 2020;73(4):408–19. 10.1080/0312407x.2019.1702707. 10.1080/0312407x.2019.1702707

[63] Noblit GW, Hare RD. Meta-ethnography: synthesizing qualitative studies. sage; 1988.

[64] Walsh D, Downe S. Meta-synthesis method for qualitative research: a literature review. J Adv Nurs. 2005;50(2):204–11. 10.1111/j.1365-2648.2005.03380.x. 15788085 10.1111/j.1365-2648.2005.03380.x

[65] Sutton J, Austin Z. Qualitative research: data collection, analysis, and management. Can J Hosp Pharm. 2015;68(3). 10.4212/cjhp.v68i3.1456. 10.4212/cjhp.v68i3.1456 PMC4485510 26157184

[66] Rankin RM, Conti J, Touyz S, Arcelus J, Meyer C, Hay P. Dancing with change: a qualitative exploration of in-session motivation to change in the treatment of anorexia nervosa. Australian Psychol. 2023;58(2):119–30. 10.1080/00050067.2022.2151338. 10.1080/00050067.2022.2151338

[67] Duncan TK, Sebar B, Lee J. Reclamation of power and self: a meta-synthesis exploring the process of recovery from anorexia nervosa. Adv Eat Disorders: Theory Res Pract. 2015;3(2):177–90. 10.1080/21662630.2014.978804. 10.1080/21662630.2014.978804

[68] Eaton CM. Eating disorder recovery: a metaethnography. J Am Psychiatr Nurses Assoc. 2019;26(4):373–88. 10.1177/1078390319849106. 31130040 10.1177/1078390319849106

[69] Espíndola CR, Blay SL. Anorexia nervosa’s meaning to patients: a qualitative synthesis. Psychopathology. 2009;42(2):69–80. 10.1159/000203339. 19225241 10.1159/000203339

[70] Fox JR, Dean M, Whittlesea A. The experience of caring for or living with an individual with an eating disorder: a meta-synthesis of qualitative studies. Clin Psychol Psychother. 2017;24(1):103–25. 10.1002/cpp.1984. 26472481 10.1002/cpp.1984

[71] Whitney J, Murray J, Gavan K, Todd G, Whitaker W, Treasure J. Experience of caring for someone with anorexia nervosa: qualitative study. Br J Psychiatry. 2005;187(5):444–9. 10.1192/bjp.187.5.444. 16260820 10.1192/bjp.187.5.444

[72] Linacre S, Green J, Sharma V. A pilot study with adaptations to the Maudsley Method approach on workshops for carers of people with eating disorders. Mental Health Rev J. 2016;21(4):295–307. 10.1108/mhrj-05-2016-0010. 10.1108/mhrj-05-2016-0010

[73] Gorrell S, Le Grange D. How best to support parents of children with an eating disorder: a commentary on Wilksch. Int J Eat Disord. 2023;56(7):1286–8. https://pubmed.ncbi.nlm.nih.gov/37184424/. 10.1002/eat.23992 PMC10759187 37184424

[74] Lonergan K, Whyte A, Ryan C. Externalisation in family-based treatment of anorexia nervosa: the therapist’s experience. J Family Therapy. 2021;44(3):351–69. 10.1111/1467-6427.12380. 10.1111/1467-6427.12380

[75] Friedlander ML, Escudero V, De Poll MJWV, Heatherington L. Meta-analysis of the alliance–outcome relation in couple and family therapy. Psychotherapy. 2018;55(4):356–71. 10.1037/pst0000161. 30335450 10.1037/pst0000161

[76] Oldershaw A, Startup H, Lavender T. Anorexia nervosa and a lost emotional self: a psychological formulation of the development, maintenance, and treatment of anorexia nervosa. Front Psychol. 2019;10. 10.3389/fpsyg.2019.00219. 10.3389/fpsyg.2019.00219 PMC6410927 30886593

[77] Fairburn CG, Shafran R, Cooper Z. A cognitive behavioural theory of anorexia nervosa. Behav Res Ther. 1999;37(1):1–13. 10.1016/s0005-7967(98)00102-8. 9922553 10.1016/s0005-7967(98)00102-8

[78] Halmi KA, Tozzi F, Thornton LM, Crow SJ, Fichter MM, Kaplan AS, et al. The relation among perfectionism, obsessive-compulsive personality disorder and obsessive-compulsive disorder in individuals with eating disorders. Int J Eat Disord. 2005;38(4):371–4. 10.1002/eat.20190. 16231356 10.1002/eat.20190

[79] Schmidt U, Treasure J. Anorexia nervosa: valued and visible. A cognitive-interpersonal maintenance model and its implications for research and practice. Br J Clin Psychol. 2006;45(3):343–66. 10.1348/014466505x53902. 17147101 10.1348/014466505x53902

[80] Pereira T, Lock J, Oggins J. Role of therapeutic alliance in family therapy for adolescent anorexia nervosa. Int J Eat Disord. 2006;39(8):677–84. 10.1002/eat.20303. 16937386 10.1002/eat.20303

[81] Isserlin L, Couturier J. Therapeutic alliance and family-based treatment for adolescents with anorexia nervosa. Psychotherapy. 2012;49(1):46–51. 10.1037/a0023905. 21967072 10.1037/a0023905

[82] Werz J, Voderholzer U, Tuschen-Caffier B. Alliance matters: but how much? A systematic review on therapeutic alliance and outcome in patients with anorexia nervosa and bulimia nervosa. Eating and Weight disorders - studies on Anorexia. Bulimia Obes. 2021;27(4):1279–95. 10.1007/s40519-021-01281-7. 10.1007/s40519-021-01281-7 PMC9079014 34374966

[83] Zaitsoff SL, Pullmer R, Cyr M, Aime H. The role of the Therapeutic Alliance in Eating Disorder Treatment outcomes: a systematic review. Eat Disord. 2014;23(2):99–114. 10.1080/10640266.2014.964623. 25330409 10.1080/10640266.2014.964623

[84] Lavender KR. Rebooting failed family-based treatment. Front Psychiatry. 2020. 10.3389/fpsyt.2020.00068. 11. 32194444 10.3389/fpsyt.2020.00068 PMC7066118

[85] Johns G, Taylor B, John A, Tan J. Current eating disorder healthcare services – the perspectives and experiences of individuals with eating disorders, their families and health professionals: systematic review and thematic synthesis. Br J Psychiatry Open. 2019;5(4). 10.1192/bjo.2019.48. 10.1192/bjo.2019.48 PMC6646967 31530301

[86] Rance N, Moller N, Clarke V. Eating disorders are not about food, they’re about life’: client perspectives on anorexia nervosa treatment. J Health Psychol. 2015;22(5):582–94. 10.1177/1359105315609088. 26446375 10.1177/1359105315609088

[87] Mansfield AK, Addis ME. Manual-based psychotherapies in clinical practice part 1: assets, liabilities, and obstacles to dissemination. Evid Based Ment Health. 2001;4(3):68–9. 10.1136/ebmh.4.3.68. 12004739 10.1136/ebmh.4.3.68

[88] Cook S, Schwartz AC, Kaslow NJ. Evidence-based psychotherapy: advantages and challenges. Neurotherapeutics. 2017;14(3):537–45. 10.1007/s13311-017-0549-4. 28653278 10.1007/s13311-017-0549-4 PMC5509639

[89] Murray SB, Rand-Giovannetti D, Griffiths S, Nagata JM. Locating the mechanisms of therapeutic agency in family-based treatment for adolescent anorexia nervosa: a pilot study of clinician/researcher perspectives. Eat Disord. 2018;26(5):477–86. 10.1080/10640266.2018.1481306. 29863443 10.1080/10640266.2018.1481306

[90] Keel PK, Dorer DJ, Franko DL, Jackson SC, Herzog DB. Postremission predictors of relapse in women with eating disorders. Am J Psychiatry. 2005;162(12):2263–8. 10.1176/appi.ajp.162.12.2263. 16330589 10.1176/appi.ajp.162.12.2263

[91] Aradas J, Sales D, Rhodes P, Conti J. As long as they eat? Therapist experiences, dilemmas and identity negotiations of Maudsley and family-based therapy for anorexia nervosa. J Eat Disorders. 2019;7(1). 10.1186/s40337-019-0255-1. 10.1186/s40337-019-0255-1 PMC6670233 31388424

[92] Couturier J, Kimber M, Szatmári P. Efficacy of family-based treatment for adolescents with eating disorders: a systematic review and meta‐analysis. Int J Eat Disord. 2012;46(1):3–11. 10.1002/eat.22042. 22821753 10.1002/eat.22042

[93] Couturier J, Lock J, Kimber M, McVey G, Barwick M, Niccols A, et al. Themes arising in clinical consultation for therapists implementing family-based treatment for adolescents with anorexia nervosa: a qualitative study. J Eat Disorders. 2017;5(1). 10.1186/s40337-017-0161-3. 10.1186/s40337-017-0161-3 PMC5582386 28878927

[94] Graham MR, Tierney S, Chisholm A, Fox JRE. The lived experience of working with people with eating disorders: a meta-ethnography. Int J Eat Disord. 2020;53(3):422–41. 10.1002/eat.23215. 31904870 10.1002/eat.23215

[95] Speers AJH, Bhullar N, Cosh S, Wootton BM. Correlates of therapist drift in psychological practice: a systematic review of therapist characteristics. Clin Psychol Rev. 2022;93:102132. 10.1016/j.cpr.2022.102132. 35316672 10.1016/j.cpr.2022.102132

[96] Dimitropoulos G, Lock J, Agras WS, Brandt H, Halmi KA, Jo B, et al. Therapist adherence to family-based treatment for adolescents with anorexia nervosa: a multi‐site exploratory study. Eur Eat Disorders Rev. 2019;28(1):55–65. 10.1002/erv.2695. 10.1002/erv.2695 PMC6925617 31297906

[97] Godart N, Berthoz S, Curt F, Perdereau F, Rein Z, Wallier J, et al. A randomized controlled trial of adjunctive family therapy and treatment as usual following inpatient treatment for anorexia nervosa adolescents. PLoS ONE. 2012;7(1):e28249. 10.1371/journal.pone.0028249. 22238574 10.1371/journal.pone.0028249 PMC3251571

[98] Agras WS, Lock J, Brandt H, Bryson SW, Dodge E, Halmi KA, et al. Comparison of 2 family therapies for adolescent anorexia nervosa. JAMA Psychiatry. 2014;71(11):1279. 10.1001/jamapsychiatry.2014.1025. 25250660 10.1001/jamapsychiatry.2014.1025 PMC6169309

[99] Cook-Darzens S, Doyen C, Mouren M. Family therapy in the treatment of adolescent anorexia nervosa: current research evidence and its therapeutic implications. Eating and Weight disorders - studies on Anorexia. Bulimia Obes. 2008;13(4):157–70. 10.1007/bf03327502. 10.1007/bf03327502 19169071

[100] Robinson AL, Dolhanty J, Greenberg LS. Emotion-focused family therapy for eating disorders in children and adolescents. Clin Psychol Psychother. 2013;22(1):75–82. 10.1002/cpp.1861. 23913713 10.1002/cpp.1861

[101] Lock J, Nicholls D. Toward a greater understanding of the ways family-based treatment addresses the full range of psychopathology of adolescent anorexia nervosa. Front Psychiatry. 2020;10. 10.3389/fpsyt.2019.00968. 10.3389/fpsyt.2019.00968 PMC6993050 32038319

[102] Tierney S. The individual within a Condition: a qualitative study of young people’s reflections on being treated for anorexia nervosa. J Am Psychiatr Nurses Assoc. 2008;13(6):368–75. 10.1177/1078390307309215. 21672876 10.1177/1078390307309215

[103] Voswinkel MMH, Rijkers C, Van Delden JJM, Elburg A. Externalizing your eating disorder: a qualitative interview study. J Eat Disorders. 2021;9(1). 10.1186/s40337-021-00486-6. 10.1186/s40337-021-00486-6 PMC8518211 34654484

[104] Cripps SC, Serpell L, Pugh M. Experiences of externalisation in recovery from anorexia nervosa: a reflexive thematic analysis. Res Sq. 2024. 10.21203/rs.3.rs-3906525/v1. 10.1186/s40337-024-01087-9 PMC11460161 39375785

[105] Abbate-Daga G, Amianto F, Delsedime N, De-Bacco C, Fassino S. Resistance to treatment and change in anorexia nervosa: a clinical overview. BMC Psychiatry. 2013;13(1). 10.1186/1471-244x-13-294. 10.1186/1471-244X-13-294 PMC3879222 24199620

[106] Cruzat־Mandich C, Díaz־Castrillón F, Escobar-Koch T, Simpson S. From eating identity to authentic selfhood: identity transformation in eating disorder sufferers following psychotherapy. Clin Psychol. 2017;21(3):227–35. 10.1111/cp.12067. 10.1111/cp.12067

[107] White M. Maps of narrative practice. 2007. http://ci.nii.ac.jp/ncid/BA81881971.

[108] White M, Denborough D, Freedman J, Epston D, White C. Narrative practice: continuing the conversations. 2011. http://www.dulwichcentre.com.au/narrative-practice-michael-white.pdf.

[109] Stillar A, Merali N, Gusella J, Scarborough J, Nash P, Orr E, Henderson K, Mayman S, Files N, Lafrance A. Caring for a child with an eating disorder: understanding differences among mothers and fathers of adolescent and adult children. Eur Eat Disorders Review: J Eat Disorders Association. 2023;31(1):87–97. 10.1002/erv.2935. 10.1002/erv.2935 35751865

[110] Duclos J, Piva G, Riquin É, Lalanne C, Meilleur D, Blondin S, et al. Caregivers in anorexia nervosa: is grief underlying parental burden? Eat Weight Disord. 2023;28(1). 10.1007/s40519-023-01530-x. 10.1007/s40519-023-01530-x PMC9941225 36807834

[111] Gorrell S, Byrne CE, Trojanowski PJ, Fischer S, Le Grange D. A scoping review of non-specific predictors, moderators, and mediators of family-based treatment for adolescent anorexia and bulimia nervosa: a summary of the current research findings. Eat Weight Disord. 2022;27(6):1971–90. 10.1007/s40519-022-01367-w. 10.1007/s40519-022-01367-w PMC9872820 35092554

[112] Bion WR. Container and contained. Group Relations Read. 1985;2(8):127–33. https://www.cpor.org/otc/Bion(1985)ContainerAndContained.pdf.

[113] Ogden TH. On holding and containing, being and dreaming. Int J Psychoanal. 2004;85(6):1349–64. 10.1516/t41h-dgux-9jy4-gqc7. 15801512 10.1516/t41h-dgux-9jy4-gqc7

[114] Johnson SM. Attachment theory in practice: emotionally focused therapy (EFT) with individuals, couples, and families. New York: Guilford; 2019. https://openlibrary.org/books/OL27341343M/Attachment_Theory_in_Practice.

[115] Robinson AL, Strahan EJ, Girz L, Wilson AE, Boachie A. I know I can help you’: parental self-efficacy predicts adolescent outcomes in family‐based therapy for eating disorders. Eur Eat Disorders Rev. 2012;21(2):108–14. 10.1002/erv.2180. 10.1002/erv.2180 22556060

[116] Hooper A, Dallos R. Fathers and daughters: their relationship and attachment themes in the shadow of an eating disorder. Contemp Family Ther. 2012;34(4):452–67. 10.1007/s10591-012-9204-8. 10.1007/s10591-012-9204-8

[117] Criscuolo M, Marchetto C, Chianello I, Cereser L, Castiglioni MC, Salvo P, et al. Family functioning, coparenting, and parents’ ability to manage conflict in adolescent anorexia nervosa subtypes. Families Syst Health. 2020;38(2):151–61. 10.1037/fsh0000483. 10.1037/fsh0000483 32525350

[118] Rousseau M, Thibault I, Blier C, Monthuy-Blanc J, Touchette L, Savard RT, et al. Intensity of family dysfunction is associated with severity of adolescent anorexia nervosa. J Family Stud. 2020;28(1):370–81. 10.1080/13229400.2020.1724817. 10.1080/13229400.2020.1724817

[119] Giles E, Cross AS, Matthews R, Lacey JH. Disturbed families or families disturbed: a reconsideration. Eating and Weight disorders - studies on Anorexia. Bulimia Obes. 2021;27(1):11–9. 10.1007/s40519-021-01160-187. 10.1007/s40519-021-01160-187 PMC8860793 33721219

[120] Cerniglia L, Cimino S, Tafà M, Marzilli E, Ballarotto G, Bracaglia F. Family profiles in eating disorders: family functioning and psychopathology. Psychol Res Behav Manage. 2017;10:305–12. 10.2147/prbm.s145463. 10.2147/prbm.s145463 PMC5633277 29042824

[121] Wallis A, Miskovic-Wheatley J, Madden S, Rhodes P, Crosby RD, Cao L, Touyz S. How does family functioning effect the outcome of family based treatment for adolescents with severe anorexia nervosa? J Eat Disorders. 2017;5(1):1–9. 10.1186/s40337-017-0184-9. 10.1186/s40337-017-0184-9 PMC5729267 29255605

[122] Dallos R. Using narrative and attachment theory in systemic family therapy with eating disorders. Clin Child Psychol Psychiatry. 2003;8(4):521–35. 10.1177/13591045030084009. 10.1177/13591045030084009

[123] Datta N, Hagan KE, Bohon C, Stern M, Kim B, Matheson BE, et al. Predictors of family-based treatment for adolescent eating disorders: do family or diagnostic factors matter? Int J Eat Disord. 2022;56(2):384–93. 10.1002/eat.23867. 36454189 10.1002/eat.23867 PMC9898138

[124] Office for National Statistics. Cost of living latest insights. 2023. https://www.ons.gov.uk/economy/inflationandpriceindices/articles/costofliving/latestinsights.

[125] National Institute of Care Excellence. Anorexia nervosa: treatment for adults. Information for the public. Eating disorders: recognition and treatment. Guidance NICE. 2017. https://www.nice.org.uk/guidance/ng69/ifp/chapter/Anorexia-nervosa-treatment-for-adults.

[126] United Nations. Definition of Youth - Factsheet. n.d. https://www.un.org/esa/socdev/documents/youth/fact-sheets/youth-definition.pdf.

[127] National Health Service Digital Service Manual. Inclusive content – Age - How to talk about different age groups and stages of life. 2021. https://service-manual.nhs.uk/content/inclusive-content/age#:~:text=child%3A%204%20to%2012%20years,18%20but%20this%20may%20vary.

[128] World Health Organisation. Pan American Health Organisation: Adolescent Health. n.d https://www.paho.org/en/topics/adolescent-health#:~:text=Adolescents%20represent%20the%20well%2Dbeing,10%20and%2024%20years%20old.

[129] Acle A, Cook BJ, Siegfried N, Beasley T. Cultural considerations in the Treatment of Eating Disorders among Racial/Ethnic minorities: a systematic review. J Cross-Cult Psychol. 2021;52(5):468–88. 10.1177/00220221211017664. 10.1177/00220221211017664

[130] Thapliyal P, Hay P, Conti J. Role of gender in the treatment experiences of people with an eating disorder: a metasynthesis. J Eat Disorders. 2018;6(1). 10.1186/s40337-018-0207-1. 10.1186/s40337-018-0207-1 PMC6088416 30123504

[131] Petticrew M, Egan M, Thomson H, Hamilton V, Kunkler R, Roberts H. Publication bias in qualitative research: what becomes of qualitative research presented at conferences? J Epidemiol Commun Health. 2008;62(6):552–4. 10.1136/jech.2006.059394. 10.1136/jech.2006.059394 18477755

[132] Higgins JPT, Thomas J, Chandler J, Cumpston M, Li T, Page MJ, Welch VA, editors. Cochrane Handbook for Systematic Reviews of Interventions version 6.4. Cochrane Database. 2023. www.training.cochrane.org/handbook. 10.1002/14651858.ED000142 PMC10284251 31643080

[133] Toye F, Seers K, Allcock N, Briggs M, Carr E, Andrews J, et al. Trying to pin down jelly’ - exploring intuitive processes in quality assessment for meta-ethnography. BMC Med Res Methodol. 2013;13(1). 10.1186/1471-2288-13-46. 10.1186/1471-2288-13-46 PMC3639821 23517438

[134] Braun V, Clarke V. Toward good practice in thematic analysis: avoiding common problems and be(com)ing aknowingresearcher. Int J Transgender Health. 2022;24(1):1–6. 10.1080/26895269.2022.2129597. 10.1080/26895269.2022.2129597 PMC9879167 36713144

